# Apoptotic Blocks in Primary Non-Hodgkin B Cell Lymphomas Identified by BH3 Profiling

**DOI:** 10.3390/cancers13051002

**Published:** 2021-02-28

**Authors:** Ryan N. Rys, Claudia M. Wever, Dominique Geoffrion, Christophe Goncalves, Artin Ghassemian, Eugene Brailovski, Jeremy Ryan, Liliana Stoica, Josée Hébert, Tina Petrogiannis-Haliotis, Svetlana Dmitrienko, Saul Frenkiel, Annette Staiger, German Ott, Christian Steidl, David W. Scott, Pierre Sesques, Sonia del Rincon, Koren K. Mann, Anthony Letai, Nathalie A. Johnson

**Affiliations:** 1 Department of Physiology, McGill University, Montreal, QC H3G 1Y6, Canada; ryan.rys@mail.mcgill.ca; 2 Lady Davis Institute for Medical Research, Jewish General Hospital, Montreal, QC H3T 1E2, Canada; claudia.wever@mail.mcgill.ca (C.M.W.); dominique.geoffrion@mail.mcgill.ca (D.G.); christophe.goncalves@mail.mcgill.ca (C.G.); artin.ghassemian@mail.mcgill.ca (A.G.); lilia_stoica@yahoo.ca (L.S.); pierre.sesques@hotmail.fr (P.S.); soniavictoria.delrincon@mcgill.ca (S.d.R.); koren.mann@mcgill.ca (K.K.M.); 3 Department of Medicine, McGill University, Montreal, QC H4A 3J1, Canada; eugene.brailovski@mail.mcgill.ca; 4 Department of Experimental Surgery, McGill University, Montreal, QC H3G 1A4, Canada; 5 Department of Medical Oncology, Dana-Farber Cancer Institute, Boston, MA 02115, USA; jeremy_ryan@dfci.harvard.edu (J.R.); anthony_letai@dfci.harvard.edu (A.L.); 6 Department of Medicine, Université de Montréal, Montreal, QC H3T 1J4, Canada; josee.hebert@umontreal.ca; 7 Division of Hematology-Oncology and Quebec Leukemia Cell Bank, Maisonneuve Rosemont Hospital, Montreal, QC H1T 2M4, Canada; 8 Division of Pathology, Jewish General Hospital, Montréal, QC H3T 1E2, Canada; tina.haliotis@mcgill.ca (T.P.-H.); svetlana.dmitrienko@mcgill.ca (S.D.); 9 Department of Otolaryngology-Head and Neck Surgery, Jewish General Hospital, Montreal, QC H3T 1E2, Canada; saul.frenkiel@mcgill.ca; 10 Department of Pathology, Robert-Bosch Hospital, 70184 Stuttgart, Germany; annette.staiger@ikp-stuttgart.de (A.S.); german.ott@rbk.de (G.O.); 11 Dr. Margarete-Fischer-Bosch-Institute of Clinical Pharmacology, University of Tübingen, 70376 Stuttgart, Germany; 12 Center for Lymphoid Cancer, BC Cancer, Vancouver, BC V5Z 1L3, Canada; csteidl@bccancer.bc.ca (C.S.); dscott8@bccancer.bc.ca (D.W.S.); 13 Departments of Medicine and Oncology, Jewish General Hospital, Montreal, QC H3T 1E2, Canada

**Keywords:** NHL, BCL2, MCL1, apoptosis, DLBCL, BH3 profiling, venetoclax

## Abstract

**Simple Summary:**

The BCL2 protein is expressed in many non-Hodgkin lymphomas (NHLs) as well as associated leukemias, e.g., chronic lymphocytic leukemia (CLL). It functions as a cell survival protein that reduces that ability of a cell to undergo mitochondrial apoptosis. However, the BCL2 inhibitor venetoclax is mainly effective in CLL, despite the expression of its protein target in NHL. We hypothesized that other mechanisms are inhibiting apoptosis in NHL: defects in pro-apoptotic signaling and/or the expression of anti-apoptotic proteins other than BCL2. Our study makes use of a technique known as BH3 profiling, which is a functional assay that determines the apoptotic competency of cells on primary NHL samples. By determining how cells in NHL avoid apoptosis upon exposure to venetoclax, we can identify patients who may benefit from additional therapies and potentially improve the response of drugs currently undergoing clinical trials for NHL.

**Abstract:**

To determine causes of apoptotic resistance, we analyzed 124 primary B cell NHL samples using BH3 profiling, a technique that measures the mitochondrial permeabilization upon exposure to synthetic BH3 peptides. Our cohort included samples from chronic lymphocytic leukemia (CLL), follicular lymphoma (FL), diffuse large B-cell lymphoma (DLBCL), high-grade B cell lymphoma with translocations in *MYC* and *BCL2* (HGBL-DH), mantle cell lymphoma (MCL) and marginal zone lymphoma (MZL). While a large number of our samples displayed appropriate responses to apoptosis-inducing peptides, pro-apoptotic functional defects, implicating BAX, BAK, BIM or BID, were seen in 32.4% of high-grade NHLs (12/37) and in 3.4% of low-grade NHLs (3/87, *p* < 0.0001). The inhibition of single anti-apoptotic proteins induced apoptosis in only a few samples, however, the dual inhibition of BCL2 and MCL1 was effective in 83% of samples, indicating MCL1 was the most common cause of lack of response to the BCL2 inhibitor, venetoclax. We then profiled Toledo and OCI-Ly8 high-grade lymphoma cell lines to determine which drugs could reduce MCL1 expression and potentiate venetoclax responses. Doxorubicin and vincristine decreased levels of MCL1 and increased venetoclax-induced apoptosis (all *p* < 0.05). Overall, in primary NHLs expressing BCL2 that have no defects in pro-apoptotic signaling, a poor response to venetoclax is primarily due to the presence of MCL1, which may be overcome by combining venetoclax with doxorubicin and vincristine-based chemotherapy or with other anti-microtubule inhibitors.

## 1. Introduction

*BCL2* is an oncogene that inhibits apoptosis [1,2]. It is expressed in many non-Hodgkin lymphomas (NHLs), including chronic lymphocytic leukemia/small lymphocytic leukemia (CLL/SLL), mantle cell lymphoma (MCL), marginal zone lymphoma (MZL), ~60% of diffuse large B-cell lymphomas (DLBCLs), and 85% of follicular lymphomas (FLs) [3]. BCL2 expression is associated with inferior survival when there is concurrent expression of MYC, an oncogene that can stimulate cellular proliferation [4,5]. This is the case in double-expressor (DE) DLBCLs and high-grade B cell lymphomas with translocations in *MYC* and *BCL2*, also known as “double-hit lymphomas” (HGBL-DH) [6]. Higher levels of BCL2 expression are associated with the presence of a *BCL2* translocation and possibly an inferior outcome in DLBCLs [7]. Venetoclax is a BCL2 homology 3 (BH3) mimetic that selectively inhibits BCL2 [8]. While effective in CLLs [9,10], venetoclax is less successful in other NHLs despite them expressing the BCL2 target [11,12]. Understanding why certain NHL cells survive after exposure to venetoclax may lead to more effective treatment regimens for these patients.

**Figure 1:**
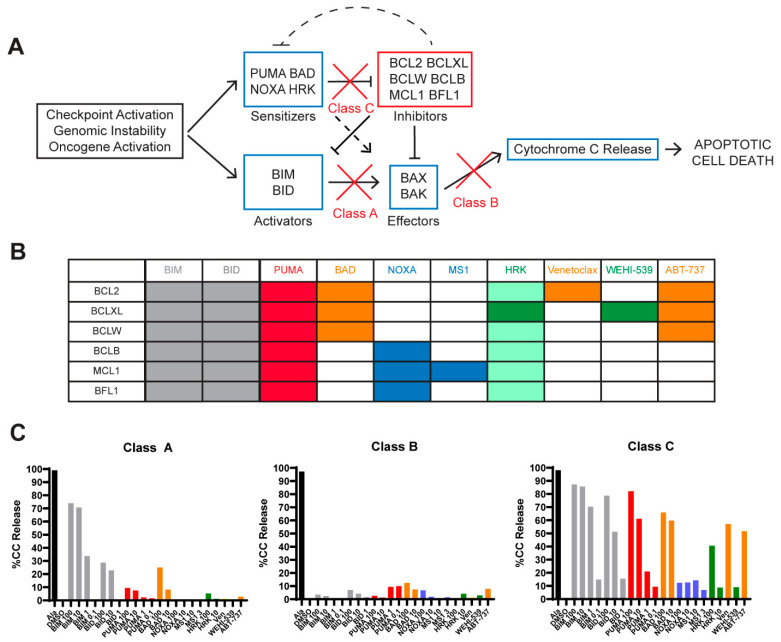
BH3 profiling assay: rationale and methodology. (Adapted from Deng et al. [16]). (**A**) Anti-apoptotic BCL2 family members inhibit the mitochondrial apoptotic pathway. Upon oncogene activation or cellular damage, activator and/or sensitizer proteins are upregulated, which allows BAX and BAK to oligomerize, leading to cytochrome c release, which results in apoptotic cell death. Anti-apoptotic BCL2 family members inhibit pro-apoptotic and sensitizer proteins, preventing BAX and BAK from inducing mitochondrial outer membrane polarization (MOMP), the commitment to apoptosis. (**B**) Pattern of interaction between the anti-apoptotic proteins (rows) present in cells and the pro-apoptotic synthetic peptides or drugs (columns) used in the BH3 profiling assay. PUMA (illustrated by red columns) inhibits all the inhibitors and is a pan-sensitizer, as well as contributing to BAX and BAK activation. Orange colors indicate peptides that inhibit the BCL2 protein with venetoclax inhibiting only BCL2 whereas BAD and ABT-737 inhibit BCL2, BCLXL, and BCLW. Blue columns are used to highlight mantle cell lymphoma (MCL)1-dependence where MS1 specifically inhibits MCL1, whereas NOXA inhibits BCLB, MCL1 and BFL1. Green columns indicate BCL-XL-dependence where WEHI-539 only inhibits BCLXL and HRK is mainly a BCL-XL inhibitor but can also inhibit other anti-apoptotic proteins with lower affinities, including BCL2. (**C**) BH3 profiles are illustrated in graphical form with % of the cells undergoing cytochrome c release on the Y axis after exposure to each drug/peptide on the X axis. Alamethecin (ALA) is a control that induces cytochrome c release in all cells independent of BAX or BAK. DMSO is the negative vehicle control because it does not induce cytochrome c release. Representative samples from our cohort were used to demonstrate different classes of apoptotic block. This high-grade B cell lymphoma with translocations in *MYC* and *BCL2* (HGBL-DH) sample shows a class A block, where cells were competent to undergo apoptosis (i.e., functional BAX/BAK) because exogenous activators BIM and BID could induce MOMP (grey bars). Two diffuse large B-cell lymphoma (DLBCL) samples shown here represent a class B and C BH3 profile. In class B blocks, cells fail to undergo MOMP upon exposure to BIM or BID BH3 synthetic peptides, indicating that there is no functional BAX or BAK, and are incompetent for undergoing apoptosis through the mitochondrial pathway. In class C blocks, cells are competent to undergo apoptosis (grey BIM/BID), primed (red PUMA) and depend mainly on BCL2 (orange bars).

Mitochondrial apoptosis is the primary mechanism of cell death following exposure to chemotherapy and cell fate lies in the balance between the pro- and anti-apoptotic BLC2 family of proteins [13]. This family of proteins share 1–4 BH domains, with BH3-only proteins being the most potent initiators of apoptosis. Upon cellular stress, “activator” BH3 proteins BIM and BID activate “effector” proteins BAX and BAK, leading to mitochondrial outer membrane permeabilization (MOMP) and subsequent cytochrome c release, an irreversible step committing the cell to undergo apoptosis. This process is inhibited by anti-apoptotic proteins (e.g., BCL2, MCL1, BCLXL, BCLW, BCLB, and BFL1), which bind to the pro-apoptotic BH3 proteins in order to prevent activation of BAX/BAK. Pro-apoptotic “sensitizer” proteins (e.g., PUMA, NOXA, BAD, and HRK), indirectly promote apoptosis by binding to anti-apoptotic proteins, thus releasing BIM/BID to activate BAX/BAK (Figure 1A). BH3 profiling assesses the functional dynamics between pro- and anti-apoptotic proteins to predict what is inhibiting mitochondrial apoptosis in live cells [14]. It uses cytochrome c release as a measure of commitment to apoptosis after exposing cells to different synthetic peptides or inhibitors, which have differing affinities for the anti-apoptotic proteins [15] (Figure 1B). BH3 profiling of DLBCL cell lines revealed three classes of apoptotic blocks, as described by Deng et al., demonstrating defects in pro-apoptotic signaling (classes A and B) or increased expression of anti-apoptotic proteins (class C) [16]. The analysis of cytochrome c release in response to BH3 peptides BIM and PUMA are key for determining the type of apoptotic block present in each sample. While BIM is a direct activator of the apoptotic effector proteins, PUMA mainly acts as a pan-sensitizer, as it broadly sequesters all types of anti-apoptotic proteins. PUMA can also directly activate BAX/BAK, but this effect has been less pronounced than BIM, especially when using BH3 domain peptides instead of full-length proteins [17,18]. Therefore, the subsequent release of cytochrome c in response to these peptides can identify whether apoptotic defects are present due to pro- or anti-apoptotic dysfunction (Figure 1, Figure S1). Class A blocks have strong responses to BIM but weak responses to PUMA, indicating the insufficient function of activator proteins to bind to BAX/BAK. Class B blocks do not respond to BIM or PUMA, have weak responses to all peptides, and indicate dysfunctional effector proteins. Class C blocks are the most common and show strong responses to several peptides, particularly to anti-apoptotic inhibition, indicating an excess of anti-apoptotic proteins as a mechanism of cell survival (Figure 1C, see supplemental methods in supplementary materials). A sample with a class C block is considered “primed” if removing the anti-apoptotic BCL2 proteins, for example with PUMA, would initiate MOMP, implying the presence of functional BIM/BID and BAX/BAK. BH3 profiling has been valuable in predicting the response to chemotherapy and BH3 mimetics in CLL [19], acute leukemias and multiple myeloma [20,21,22]. It has also predicted the response to BH3 mimetics in DLBCL cell lines [16] but has not yet been reported in primary NHLs due to the lack of archived cells frozen as viable cell suspensions.

We hypothesized that BCL2+ NHLs that do not respond to venetoclax have defects in pro-apoptotic signaling or express other anti-apoptotic proteins not targeted by venetoclax. Our aims were to use BH3 profiling to determine the apoptotic blocks present in primary NHLs and to determine whether exposure to chemotherapy agents could identify drugs that could synergize with venetoclax and maximize cell death. We found that NHLs have mostly class C blocks, depending mainly on BCL2 and MCL1 for survival, but we also discovered a subset of samples displaying clear pro-apoptotic protein defects. Finally, we found that pre-treatment with doxorubicin and vincristine sensitizes DLBCL cell lines to venetoclax.

## 2. Results

### 2.1. BH3 Profiling Is Reproducible in Primary Cells

**Figure 2:**
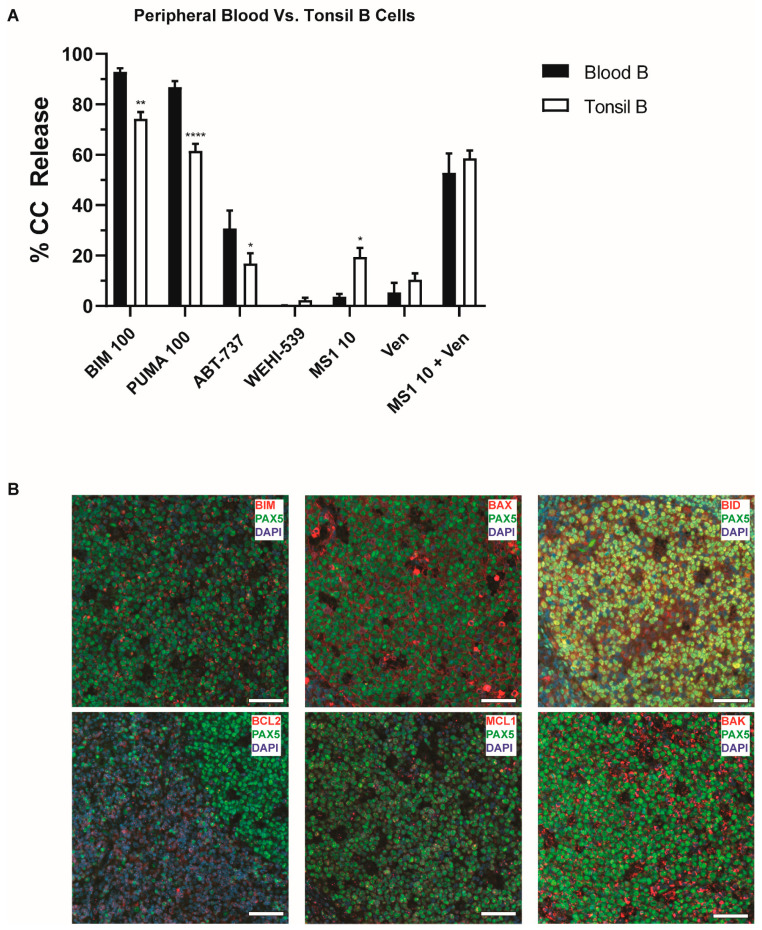
BH3 profiling of peripheral blood (PB) and tonsil B cells. (**A**) The percentage of cells undergoing cytochrome c release is displayed on the *y*-axis according to different cell subsets identified on the *x*-axis. Shown is the comparison of frozen B lymphocytes from tonsils and peripheral blood. Tonsillar B cells have lower responses to BIM and PUMA, while showing increased responses to MS1. Bars represent the mean ± standard error of the mean (SEM). Analysis was carried out using a 2-way ANOVA comparing peptide exposure or subtype with cytochrome c release. * *p* < 0.05, ** *p* < 0.01, **** *p* < 0.0001. (**B**) Representative merged immunofluorescence (IF) images of normal tonsil. Images were taken on a 20× objective highlighting staining of DAPI (blue), PAX5 (green, AF594) and the protein of interest (red, AF647). Scale bars represent 50 µm. Pro-apoptotic proteins are expressed in normal germinal center (GC) cells, along with MCL1. BCL2 expression is confined to the edges of the GC follicle and PAX5 negative cells.

We first established the reproducibility of BH3 profiling and physiologic responses to apoptotic stimuli using peripheral blood (PB) and tonsillar B cells from 24 different healthy individuals. Normal B cells had high cytochrome c responses to BIM and PUMA (>60%), with PB B cells having a much lower coefficient of variation (CV) (5.1% for BIM, 8.4% for PUMA) than tonsillar B cells (13.6% for BIM, 17.2% PUMA). The increased variability in tonsils may reflect a differing biology and the increased manipulation of these cells when generating a cell suspension. Considering that the majority of our samples are derived from patient lymph nodes, we cannot rule out these contributions to any variability in responses. For those reasons, tonsillar B cells were used to establish thresholds to define class A/B blocks, as it reflects the largest part of our cohort. Low cytochrome c responses (0–30%) were defined as being greater than 2.5 standard deviations away from the lowest mean response in tonsillar B cells, with no normal B cell (PB or tonsil) falling into this range (Figure 2A, Figure S2). We also measured cytochrome c release in DLBCL cell lines and demonstrated that cytochrome c release by BH3 profiling correlates with cell viability at 17 h (Figure S1B). Cell viability studies in primary cells proved to be ineffective, as there were insufficient cells to run subsequent experiments and/or poor viability for these cells under standard culture conditions. However, the results from our cell lines support that cytochrome c release after MOMP is an early commitment to apoptosis. The summary of all BH3 profiles is provided in Figures S3 and S4.

### 2.2. Normal B Lymphocytes Are Partially Dependent on BCL2

We then compared the BH3 profiles of PB and tonsillar B cells to determine if there were any differences in “priming” or dependence on anti-apoptotic proteins. Tonsillar B cells had lower responses to PUMA and BIM (all *p* < 0.001), suggesting they were more resistant to apoptosis and less “primed” for cell death than PB B cells. Both groups had relatively low responses to individual anti-apoptotic protein inhibition, except that tonsillar B cells had higher responses to MS1 (*p* < 0.05), suggesting they were more MCL1-dependent (Figure 2A). Much higher cytochrome c responses were achieved when combining inhibitors to both BCL2 and MCL1. Immunofluorescence staining for PAX5, a B cell transcription factor used for identification [23], and intrinsic apoptotic proteins was carried out on tonsil tissue to establish the expression of these proteins, as previously reported [24,25,26]. Germinal center (GC) cells expressed BAX, BIM, BID, and BAK, while MCL1 was expressed in multiple punctae and BCL2 was preferentially expressed outside the GC (Figure 2B). Taken together, normal B lymphocytes had typical class C profiles, were primed, and dependent on BCL2 and MCL1 for survival.

### 2.3. Defects in Pro-Apoptotic Signaling Are a Feature of High-Grade Lymphomas and Poor Responses to Venetoclax

**Figure 3:**
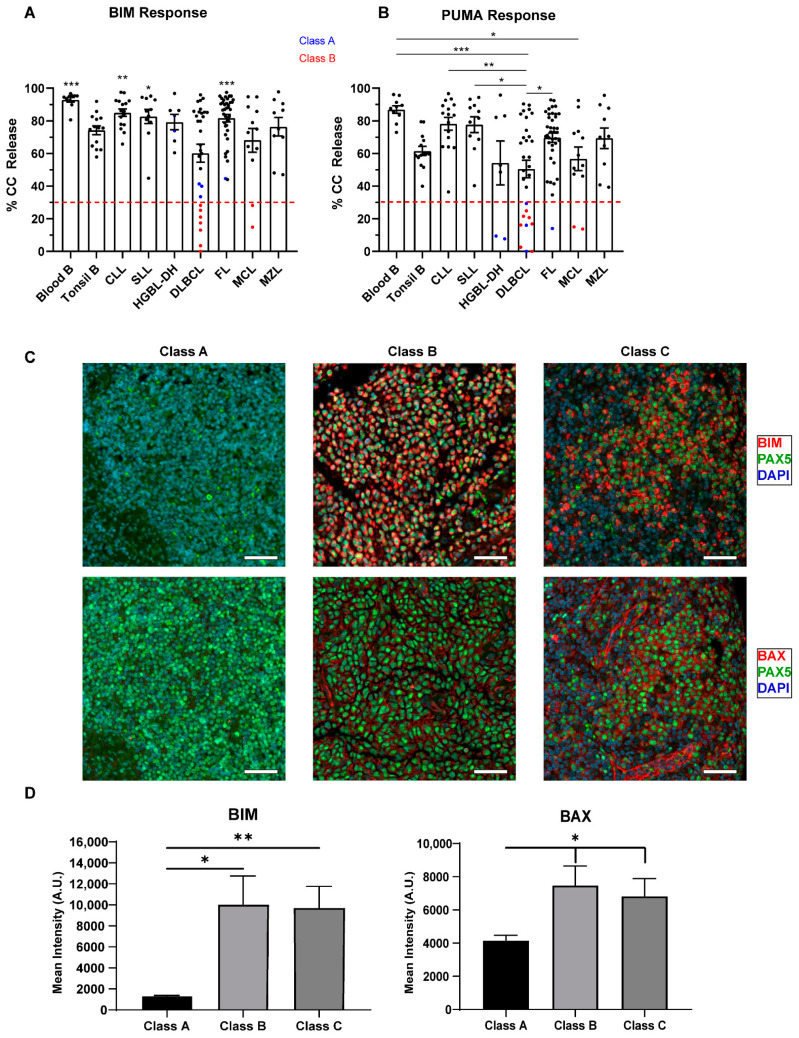
Competency and priming of the mitochondrial pathway according to lymphoma subtype. We measured cytochrome c release, displayed on the *y*-axis, after exposure to 100 μM of BIM (**A**) and 100 μM of PUMA (**B**) peptides in different untreated lymphoma subsets (10 frozen blood B, 14 tonsil B, 16 chronic lymphocytic leukemia (CLL), 11 small lymphocytic leukemia (SLL), 7 high-grade B cell lymphomas with translocations in *MYC* and *BCL2* (HGBL-DH), 30 DLBCL, 38 follicular lymphoma (FL), 12 mantle cell lymphoma (MCL), 10 marginal zone lymphoma (MZL)). For BIM, we defined a threshold of <30% as having a class B block, i.e., having dysfunctional BAX or BAK. For PUMA, we defined a threshold of <30% as being unprimed. Red dashed lines highlight the 30% cut-off, with samples below this threshold being class A/B apoptotic blocks. Class A block samples are in blue and class B block samples are in red. Dots represent individual samples, bars represent the mean ± standard error of the mean (SEM). A one-way ANOVA with Tukey multiple comparison was used to determine statistical significance between non-Hodgkin lymphoma (NHL) subtypes. Only comparison to DLBCL was determined to be significantly different in BIM response. DLBCL samples are less primed than other NHL and high-grade lymphomas have a higher percentage of class A/B blocks. * *p* < 0.05, ** *p* < 0.01, *** *p* < 0.001. (**C**) Representative merged IF stains at 20× for DAPI (blue), PAX5 (green, AF594) and BIM/BAX (red, AF647) in class A, B, and C DLBCL samples. Scale bars represent 50 µm. (**D**) Quantification of the mean pixel intensity across all classes of apoptotic block in DLBCL. Samples were analyzed using Qupath software and t-test with Welch’s correction was used to compare columns, bars represent the mean ± standard error of the mean (SEM). * *p* < 0.05, ** *p* < 0.01. Class A samples may have decreased expression of BIM and possibly BAX compared to Class B and C samples.

**Figure 4:**
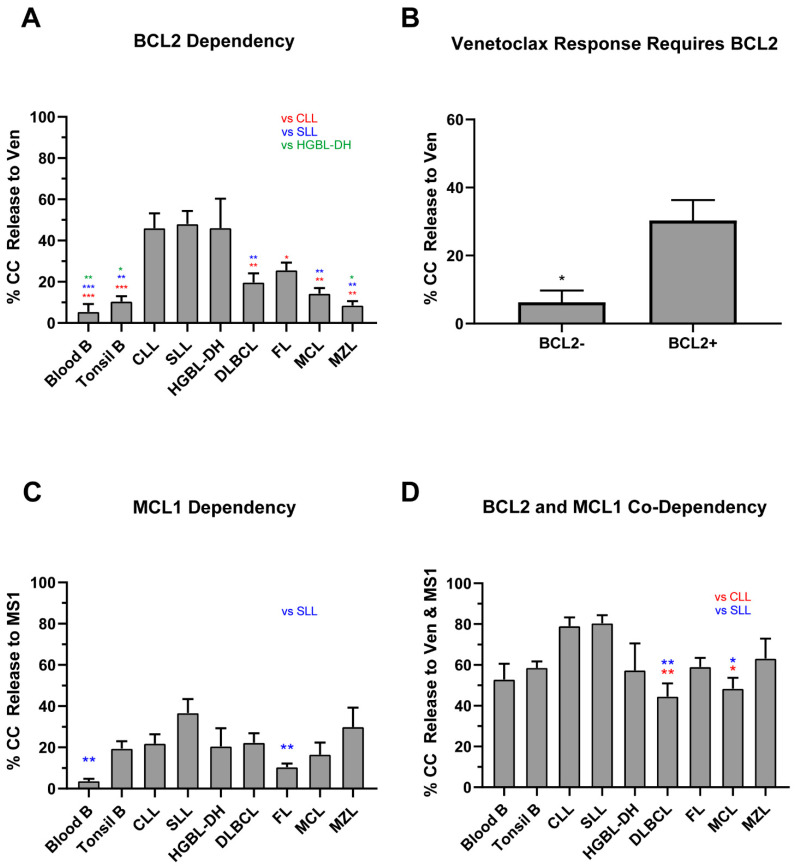
Sensitivities to BCL2 and MCL1 inhibition according to lymphoma subtype. We measured cytochrome c release, displayed on the *y*-axis, after exposure to 1 μM of venetoclax and 10 μM of MS1 in different lymphoma subsets (*x*-axis). A one-way ANOVA with Tukey multiple comparison was used to determine statistical significance between NHL subtypes. Bars represent the mean ± standard error of mean (SEM). (**A**) Comparison of lymphoma subtypes based on venetoclax response. CLL, SLL, and HGBL-DH showed notable responses to BCL2 inhibition. (**B**) Venetoclax responses in DLBCL and HGBL-DH samples according to BCL2 protein expression (present in >50% of cells). Venetoclax responses were not seen in high-grade NHL in the absence of BCL2. T-test with Welch’s correction was applied to assess significance. (**C**) MS1 responses in lymphoma subtypes. SLL displays the greatest response to single MCL1 inhibition. (**D**) NHL response to dual inhibition of BCL2 and MCL1 by venetoclax and MS1. All subtypes display increased responses when compared to singular inhibition. * *p* < 0.05, ** *p* < 0.01, *** *p* < 0.001. Abbreviations: CLL, chronic lymphocytic leukemia; FL, follicular lymphoma; DLBCL, diffuse large B cell lymphoma; HGBL-DH, high grade B cell lymphoma with translocations in *MYC* and *BCL2*; MCL, mantle cell lymphoma; MZL, marginal zone lymphoma.

Low grade NHLs (FL, CLL, MZL and MCL) had class C blocks, as determined by responses to BIM and PUMA, with similar profiles to normal B cells (Figure 2 and Figure 3). Although we were limited to 12 samples, we showed that MCL had significantly lower PUMA responses compared to PB B cells (*p* = 0.0320). CLL and SLL are generally considered to be different representations of the same disease, their main difference being that the malignant cells arise from either the blood/bone marrow (CLL) or the lymph nodes (SLL). Comparing CLL/SLL cells obtained from different compartments, PB versus lymph node (LN), SLL cells had similar responses to BIM and PUMA but higher responses to MS1, indicating a more prominent MCL1 dependence in the LN, similar to our tonsillar controls (Figure 4C). Overall, low-grade NHLs are primed and would undergo apoptosis except for the presence of anti-apoptotic proteins.

DLBCL had significantly lower responses to both BIM and PUMA (*p* = 0.0002 for both, Figure 3A) compared to normal lymphocytes, suggesting that high-grade NHLs tend to have dysfunctional pro-apoptotic signaling. DLBCL displayed a wider range of responses to both peptides when compared to controls and other subtypes (BIM CV: 50.3%, PUMA CV: 58.3%), reflecting the heterogeneous nature of this disease. Class B blocks were observed in nine samples: seven DLBCL and two MCL and class A blocks in six samples: three DLBCL, two HGBL-DH, and one FL. Class A and B blocks were detected in 32.4% of cases with high-grade histology (12/37) compared to 3.4% of low-grade lymphomas (3/87, *p* < 0.0001). Data on clinical outcome were available in 24/37 of the high-grade cases, with 67% (16/24) of cases experiencing a relapse, suggesting that class A/B blocks may be associated with chemo-resistance. We hypothesized that the functional defects in these cases as shown by BH3 profiling is a result of reduced expression or the absence of pro-apoptotic proteins. Additionally, formalin-fixed paraffin embedded (FFPE) tumor tissue was available for 15 DLCBL patients that underwent BH3 profiling (two class A, five class B, eight class C). The expression of BIM and BAX was assessed by immunofluorescence dual staining in all three classes of apoptotic block (Figure 3B,C, Table S2). The mean intensity values for BIM and BAX showed a reduced expression in the two class A samples, indicating that the reduction of these proteins may be a contributing factor to their apoptotic defects and reduced response to BH3 mimetics. These observations would need to be validated on a larger sample set. Interestingly, class B samples had a similar expression of BAX compared to apoptotic competent class C samples. We did see a reduction in the expression of BIM in class B samples, but not to the extent seen in class A (*p* = 0.1554). Additional staining for BAK, BID, and BCL2 revealed similar expression levels across all classes of apoptotic blocks (Figures S5 and S6). Overall, our data suggest that class B samples have the pro-apoptotic proteins necessary to initiate cell death, but there is an additional mechanism at play which inhibits these cells from releasing cytochrome c.

### 2.4. Lack of BCL2 Expression and MCL1 Dependency Result in a Poor Venetoclax Response in Class C B-NHLs

Since 88% (109/124) of B-NHLs had class C blocks, we first determined how many samples were primarily BCL2-dependent. As expected, CLL and SLL had the greatest responses to venetoclax, significantly higher than all other subtypes except for HGBL-DH (all *p* < 0.05, Figure 4A). In fact, 70% of CLL/SLL and 57% HGBL-DH (19/27 and 4/7, respectively) displayed BCL2-dependency, versus only 21% (19/90) for all other NHLs (*p* < 0.0001 and *p* = 0.0310, respectively, response defined as ≥30% cytochrome c release). We then assessed the venetoclax response in DLBCL stratified by BCL2 protein expression via clinical immunohistochemistry at diagnosis (see supplemental methods in supplementary materials). A lack of BCL2 protein expression in primary high-grade lymphomas, i.e., BCL2- negative defined as being present in <50% of cells, was associated with a very low venetoclax response (the mean response was 30.3% for BCL2+ and 6.3% for BCL2-negative, *p* = 0.0371, Figure 4B). The cell of origin subtype or presence of *BCL2* translocations were not associated with venetoclax response (Table S1). These data support previous findings that venetoclax is ineffective in the absence of its target, BCL2 protein [27].

**Figure 5:**
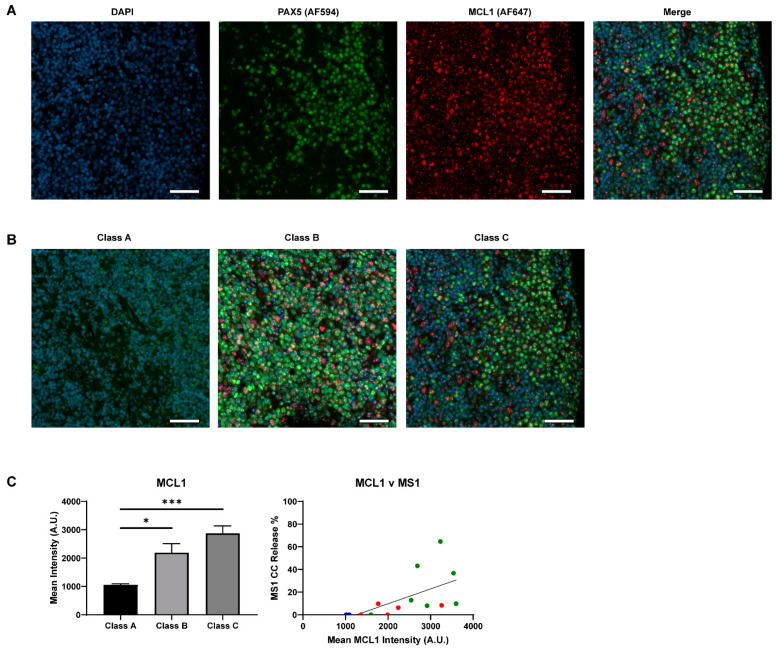
Expression of MCL1 in DLBCL by immunofluorescence. (**A**) Representative 20× single channel images of DAPI (blue), PAX5 (green, AF594), and MCL1 (red, AF647), as well as a merged image, in a class C sample. Scale bars represent 50 µm. (**B**) Qualitative comparison of MCL1 expression in class A, B, and C samples. The same sample from panel A is used in this panel. PAX5 staining was weak in class A when co-stained with MCL1, but this tissue was confirmed to be PAX5 positive in previous stains. Scale bars represent 50 µm. (**C**) The mean pixel intensity of all samples in each class, as calculated by Qupath. T-test with Welch’s correction was used to compare columns, bars represent the mean ± standard error of the mean (SEM). Spearman linear regression analysis was applied to analyze the correlation between cytochrome c release to MS1 (10 µM) and mean MCL1 intensity (*R^2^* = 0.3525). Dot colors represent class of sample: A (blue), B (red), C (green). Response was only seen in class C samples with high MCL1 expression and mean MCL1 expression was lower in Class A/B samples * *p* < 0.05, *** *p* < 0.001.

MCL1 was the second most common dependency in NHLs. The mean responses to the MCL1 specific inhibitor MS1 were lower than venetoclax with the highest responses (30%–40%) observed in SLL and MZL (Figure 4C). Low responses (15–30%) were detected in CLL, DLBCL, MCL and HGBL-DH and the lowest responses were in seen in FL (10%). Interestingly, 6/30 of the DLBCL samples and 3/10 of the MZL samples profiled showed an MCL1-dominant response, with no response to BCL2 inhibition. Dual-IF of MCL1 in our FFPE DLBCL (Figure 5A,B) samples showed that the responders to MS1 had relatively high expression of the protein and that overall increased expression trended with increased cytochrome c release by BH3 profiling (Figure 5C). Similar to the on-target engagement of venetoclax with BCL2, our data support the necessary expression of the target MCL1 for a successful response to targeted inhibition.

Given the modest responses to single peptides, we speculated that more than one anti-apoptotic protein was involved in inhibiting apoptosis in class C NHLs. The individual responses to MCL1 in our own samples, and the published data supporting that MCL1 inhibits apoptosis in DLBCL [27,28] and AML/NHL cell lines [29,30], prompted us to test the co-dependency of BCL2 and MCL1 in a subset of our NHLs. MCL, CLL, FL, and DLBCL samples all showed significant increases in cytochrome c release when using both venetoclax and MS1 together in the BH3 profiling assay, compared to single agents venetoclax or MS1 (all *p* < 0.05, Figure 4A,C,D). CLL and SLL displayed the greatest response to combination therapy, with the levels of cytochrome c release significantly greater when compared to DLBCL (*p* = 0.0014 and 0.0011, Figure 4D). In fact, the levels of cytochrome c release were similar to those seen with PUMA, which inhibits all anti-apoptotic proteins, suggesting the contribution of a third anti-apoptotic protein beyond BCL2 and MCL1 is minimal. HGBL-DH had similar responses to dual inhibition as with venetoclax alone (*p* = 0.84) but most of the HGBL-DH in our dataset were obtained from PB and marrow, so we cannot exclude that HGBL-DH obtained from LN compartments would have more MCL1 co-dependence, as was the case with tonsils and nodal SLL. Overall, we note that the dual inhibition of MCL1 and BCL2 was effective in 83% of NHLs.

**Figure 6:**
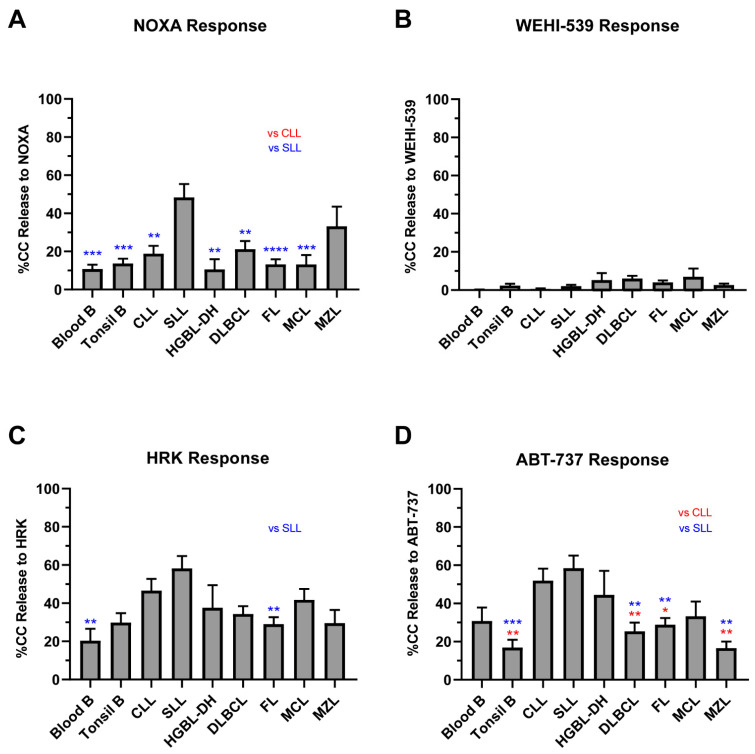
BCLXL and other anti-apoptotic protein dependency. Graphs show cytochrome c release after 90-min exposure to 100 μM of NOXA (**A**), 1 μM of ABT-737 (**B**), 1 μM of WEHI-539 (**C**), and 100μM of HRK (**D**) in all NHL subtypes. Bars measure the mean of all samples; error bars indicate standard error of the mean (SEM). A one-way ANOVA with Tukey multiple comparison was used to determine statistical significance between subtypes. NHLs respond poorly to the singular inhibition of anti-apoptotic proteins but respond better when multiple proteins are targeted. BCLXL contributes only modestly to cell survival in our cohort. * *p* < 0.05, ** *p* < 0.01, *** *p* < 0.001, **** *p* < 0.0001.

The evaluation of additional anti-apoptotic proteins was carried out to verify our hypothesis that BCL2 and MCL1 were the primary causes of apoptotic block in primary NHL. NOXA, an MCL1, BCLB and BFL1 inhibitor, had low responses in all subtypes except for SLL (Figure 6A). However, the NOXA responses were similar to the MS1 responses, indicating the low contribution of BCLB and BFL1 to cell survival. BCLXL dependency was low across all subtypes, with the mean WEHI-539 responses being <10% and very few samples showing a response (Figure 6B). HRK, another BCLXL inhibitor, displayed more modest responses in NHL but this is likely due to the fact that it also has low affinity binding for other anti-apoptotic proteins (Figure 6C). Responses to ABT-737 and venetoclax were similar, suggesting the effect of ABT-737 was mainly through BCL2 inhibition, not BCL-XL. Similar to MS1, we tested the combination of WEHI-539 and venetoclax in a subset of FL and DLBCL profiles (Figure S7). There was no significant increase in the release of cytochrome c with the additional targeting of BCLXL by WEHI-539 compared to venetoclax alone. Thus, our data argue against BCLW or BCLXL being significant contributors to poor venetoclax responses in primary NHLs (Figure 6D).

In summary, the BH3 profiling of primary NHLs shown here supports at least three causes of poor venetoclax responses: pro-apoptotic signaling defects, a lack of BCL2 protein expression in high-grade lymphomas, as well as co-dependence on MCL1, observed in all NHL subtypes.

### 2.5. Dynamic BH3 Profiling (DBP) Reveals Drugs That Can Synergize with Venetoclax

**Figure 7:**
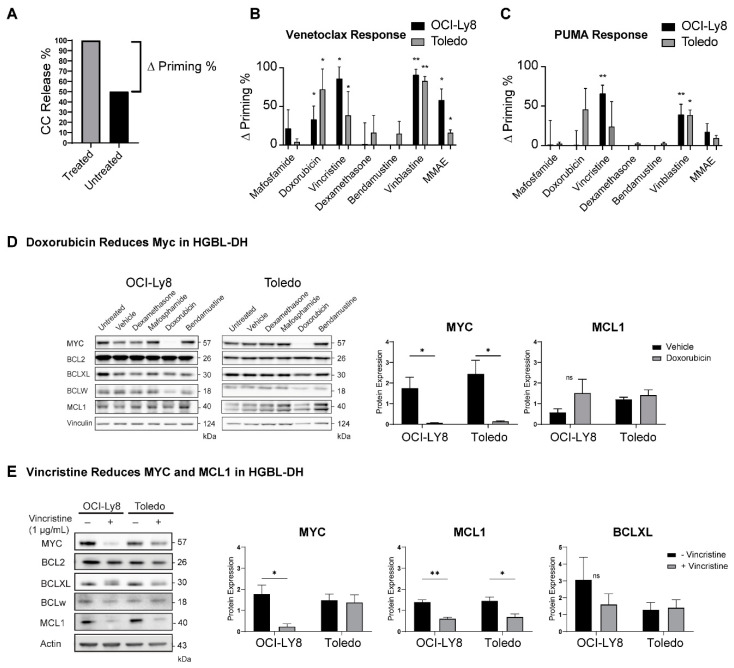
Dynamic BH3 profiling performed in Toledo and OCI-Ly8 cell lines. The % of untreated cells undergoing cytochrome c release was subtracted from the % of cells undergoing cytochrome c release in treated samples to determine an increase in release named “Δ Priming” (example shown in panel (**A**)). Cytochrome c release was measured in cells upon exposure to 1 µM of venetoclax (**B**) and 1 µM PUMA (**C**) after a 17 h incubation with dimethyl sulfoxide (control) and different drugs. Welch’s *t* tests were used to compare individual treatments to the individual control groups. Microtubule targeting components of RCHOP such as doxorubicin and vincristine increased cells priming and sensitivity to venetoclax. *p < 0.05, **p < 0.01. (**D**) Western blot measuring anti-apoptotic protein and MYC levels in OCI-Ly8 and Toledo cell lines after 17 h treatment with dexamethasone, mafosphamide, doxorubicin, and bendamustine. Doxorubicin significantly decreases the expression of MYC in both cell lines after treatment. (**E**) Western blot measuring anti-apoptotic protein and MYC levels in OCI-Ly8 and Toledo cell lines after 17 h treatment with vincristine. Bars measure the mean of all samples, error bars indicate SEM. Vincristine significantly reduces MCL1 expression in both cell lines while also reducing MYC expression in OCI-LY8. BCLXL was not significantly reduced after treatment with vincristine. * *p* < 0.05, ** *p* < 0.01. ns, non-significant.

Given that venetoclax is being tested in combination with other chemotherapies, such as rituximab, cyclophosphamide, doxorubicin, and vincristine (components of RCHOP) in DLBCL [31] and bendamustine-rituximab in FL [32], we determined whether exposure to frontline chemotherapy drugs could bring cells closer to the apoptotic threshold and synergize with venetoclax to initiate apoptosis. Since the supply of primary lymphoma cells is limited, and their growth and viability are poor ex vivo over long periods of culture, we performed BH3 profiling on HGBL-DH cell lines after exposure to different chemotherapies in vitro. The change in priming (Δ priming %) was calculated by subtracting the untreated response from the treated response as determined by BH3 profiling. (Figure 7A). Mafosphamine (cyclophosphamide), dexamethasone, and bendamustine did not prime the cells or sensitize them to venetoclax. Within components of RCHOP, doxorubicin and vincristine significantly increased cellular responses to venetoclax (Figure 7B), a feature that was shared with other microtubule-targeting drugs, such as vinblastine and monomethyl auristatin E (MMAE). Vincristine and vinblastine increased the cells’ priming, as measured by increased responses to 1 µM of PUMA peptide, suggesting these may be effective in correcting class A blocks (Figure 7C).

We then evaluated whether chemotherapy could change the levels of anti-apoptotic proteins or MYC in DLBCL. We hypothesized that the concentration of proteins with the shortest half-lives, such as MYC [33] and MCL1 [34], may decrease after exposure to drugs that cause cell cycle arrest and/or inhibit transcription [35]. Maphosphamide, dexamethasone, or bendamustine did not change the levels of anti-apoptotic proteins or MYC in these DLBCL cell lines. Doxorubicin exposure decreased MYC but had no effect on the levels of anti-apoptotic proteins (Figure 7D). Vincristine had the most dramatic effect on cells by decreasing MYC in OCI-Ly8, and MCL1 levels in both cell lines, without significantly changing the levels of other proteins (Figure 7E). Levels of BIM remained constant in treated samples, indicating that vincristine increases priming by reducing the availability of MCL1, as opposed to activating BIM (Figure S8). Therefore, doxorubicin and microtubule-targeting drugs might synergize with venetoclax in NHL that overexpress MYC, BCL2 and MCL1. Overall, this suggests that combining RCHOP or other microtubule inhibitors with venetoclax could be effective in BCL2+ NHLs. This strategy may also help in correcting class A blocks, as shown by increases in overall priming, but would not be effective in overcoming class B blocks.

## 3. Discussion

The overexpression of BCL2 protein is a common mechanism of inhibiting apoptosis in NHL. We used BH3 profiling to study the mitochondrial apoptotic pathway in viable primary NHL cells and determine which BH3 proteins are keeping the cells alive. Our three main findings are that a lack of a venetoclax response is primarily a consequence of pro-apoptotic protein dysfunction, the absence of BCL2 protein and the presence of MCL1, the initial finding being a novel feature of lymphomas with high-grade morphology. This study provides original insights into apoptotic resistance in rare primary NHL samples that could not be extrapolated from immortalized cell lines. Based on our dynamic BH3 profiling results, venetoclax would have synergistic effects with microtubule inhibitors and doxorubicin. Overall, this new knowledge may help tailor treatment regimens for patients with B-NHLs and subsets of CLL patients, such as those presenting with Richter’s transformation or those receiving ibrutinib, which may synergize with venetoclax by reducing expression of MCL1 and BCLXL [36,37].

This study improves our understanding of the apoptotic dependencies of normal B cells in both PB and tonsils. While normal PB B lymphocytes express BCL2, their dependency on MCL1 relatively protects them from venetoclax-induced apoptosis. This work may also provide some insight into why venetoclax may be more active in clearing lymphoma cells from the PB compared to nodal compartments [38]. Compared to tonsillar B cells, PB B cells were significantly more primed, closer to the apoptotic threshold and less dependent on MCL1. This may in part be attributed to B cells that are at different stages of differentiation, since BCL2+ naïve and memory B cells are found predominantly in the PB compartment, whereas tonsils contain germinal center B cells, which are known to be BCL2-negative and MCL1-dependent [26,39,40,41]. It is also possible, however, that factors within the tissue microenvironment affect priming or dependency on other anti-apoptotic proteins, notably MCL1 [42]. Thus, the higher MCL1 dependency in LN compartments may be a source of early relapse in CLL patients treated with single agent venetoclax.

The profound pro-apoptotic defects observed in a subset of DLBCLs indicates a mechanism of resistance to venetoclax, and possibly chemotherapy, that is under-appreciated. This feature was especially prevalent in HGBL-DH and DLBCL, suggesting an association between pro-apoptotic defects, high-grade lymphomas, and clinical resistance to conventional RCHOP chemotherapy or drugs targeting apoptosis. Low responses to BIM and PUMA imply that the effector proteins BAX/BAK are not functioning correctly. Anti-apoptotic proteins can bind directly to BAX/BAK but low responses to drugs targeting these proteins suggests that the problem lies with dysfunctional pro-apoptotic proteins or the subsequent steps prior to cytochrome c release. Our protein expression data imply that these proteins are expressed, except for class A samples, but more samples are needed for verification. The nature of this dysfunction warrants further exploration, as some studies have indicated that apoptosis can proceed in some cancer cell lines treated with BH3 mimetics without functional BH3-only proteins such as BIM and PUMA [43]. Therefore, methods independent of BH3-mediated apoptosis may also be involved and contribute to class A/B dysfunction. This also suggests that finding alternative means of triggering cell death that are outside of mitochondrial apoptosis may be beneficial to patients with low responses to any combination of BH3 mimetics. Studying additional samples of high-grade morphology would be important to further understand the relationship between the functional defects in apoptosis (phenotype) and their underlying genomic alterations (genotype). We recently reported a class B block in a patient with relapsed Burkitt lymphoma, where a mutation in *BAX* resulted in no production of BAX protein [44]. Mutations in *BAX* or *BAK,* however, are not a typical feature of de novo or relapsed DLBCL [45]; therefore, it is more likely that the class B phenotype seen in our samples arose by a different mechanism. Overall, these data suggest that BH3 profiling may be a useful technique to identify patients that could benefit from therapies that kill lymphoma cells independent of the mitochondrial pathway. Such therapies include immunotherapies that initiate death via cell-mediated or complement mediated cytotoxicity. Examples include chimeric antigen receptor T (CAR-T) cell regimens, bi-specific T cell engagers (BiTEs), immune check-point inhibitors or other immunomodulating agents [46,47,48,49].

The main cause of a lack of venetoclax response in B-NHLs in our study is the presence of MCL1. Due to the rarity of primary viable NHL samples, the number of samples studied is limited. That said, we provide the largest analysis of primary NHL samples by BH3 profiling to date, highlighting the relative contribution of each potential cause of poor venetoclax responses according to the NHL subtype. Dependency on BCL2, MCL1 and BCLXL has recently been reported in NHL cell lines [50,51], but we did not find a significant contribution for BCLXL in our primary NHL samples. Given that NHL, specifically DLBCL, is such a heterogenous disease, we cannot rule out that other anti-apoptotic proteins contribute to tumor cell survival in minor subsets of patients if a larger number of samples were analyzed. There is some evidence that primary treatment with RCHOP may influence anti-apoptotic protein dependency [51]. Our samples are all from untreated patients, therefore it may be that anti-apoptotic protein dependencies other than BCL2 and MCL1 will exist in this population after treatment or at relapse. Co-expression of BCL2 and MCL1 has been reported in NHLs [12,52,53,54] and MCL1 dependency is also seen in acute myelogenous leukemia and multiple myeloma [55,56]. In lymphomas, MCL1 dependence is likely inherited from the normal B cell counterpart and by being within the microenvironment of the lymph node compartment [57,58]. In DLBCL cell lines, *NOXA* amplification or MCL1 inhibition has sensitized DLBCL to venetoclax, but only when BIM is present [27]. Treatment with venetoclax and MS1 greatly increased the apoptotic response of primary patient samples and appeared synergistic in some cases. Our data confirm prior reports that MCL1 protein levels decrease following exposure to vincristine [59,60]. In fact, three different microtubule inhibitors sensitized cells to venetoclax, suggesting that this is a class effect. RCHOP and venetoclax should be effective in high-grade lymphomas that express BCL2 protein, are not class B, and co-depend on BCL2 and MCL1 (53% of our diagnostic DLBCL samples). This combination appeared to translate into a clinical benefit in patients with BCL2+ DLBCL in the phase II CAVALLI study [31]. Thus, BH3 profiling could be used to predict patients’ responses to venetoclax and select drugs that could be synergistic. Recently, a high-throughput BH3 profiling methodology was developed to screen the efficacy of numerous drugs on inducing apoptosis in malignant cells [61].

Placed into a clinical context, the BH3 profiling of primary lymphoma samples has allowed us to gain insight into why there is such great variability in clinical responses to venetoclax in NHLs. Our results mirror those obtained in clinical trials, where CLL is the most venetoclax-responsive NHL, while FL, MCL, and DLBCL have more modest responses. While the BH3 profiling assay in our study is relatively easy to apply, its main limitation is the requirement of a large number of live malignant cells (5–10 million) in a cell suspension. In cases where enough cells are available, BH3 profiling could be applied clinically to identify patients who would benefit from conventional chemotherapy (class C), targeted therapies such as venetoclax, or are candidates for alternative immunotherapies (class B). Based on our dynamic BH3 profiling results, combinations using venetoclax and microtubule agents with or without anthracyclines would be effective in NHLs having class A or C blocks. Thus, adding venetoclax to RCHOP-like regimens or to the MMAE-conjugated anti-CD79b antibody polatuzumab [62,63] may be strategies to overcome the chemo-resistance associated in a subset of patients with BCL2+ high-grade lymphomas. The lack of venetoclax responses in BCL2-negative DLBCL supports the notion that the presence of BCL2 protein is required to obtain a response to venetoclax, and that patients who have BCL2-negative lymphomas may not benefit from venetoclax-based regimens. Our report of pro-apoptotic defects in DLBCL and HGBL-DH, as seen by the increased frequency of class A/B samples, indicates a mechanism of resistance to venetoclax and possibly chemotherapy that has yet to be sufficiently explored and warrants future studies into the possible rescue of these defective proteins.

## 4. Materials and Methods

### 4.1. Sample Acquisition and Preparation

For this project, we profiled 148 samples: 111 samples were obtained at the Jewish General Hospital in Montreal, 12 from the Banque de cellules leucémiques du Québec (BCLQ), 13 from the British Columbia Cancer agency, and 23 from Robert-Bosch Hospital, Stuttgart, Germany. This project was approved by the Research Ethics Board protocols [11–047 and 12–052]. Of these 148 samples, 124 were NHL: 16 CLL, 11 SLL, 38 FL, 29 DLBCL, 7 HGBL-DH, 12 MCL, 10 MZL and 1 HGBL without translocations in *MYC* and *BCL2,* which was included within the DLBCL category in Table S1. All samples were taken prior to chemotherapy (*n* = 124). Normal controls included B cells from 14 tonsils and 10 PB samples. All cells were obtained from disaggregated tissue cell suspensions, blood, or fluids and viably cryopreserved using protocols outlined in the supplemental methods in supplementary materials. We also used HGBL-DH and DLBCL cell lines SUDHL10, Toledo, and OCI-Ly8; generous gifts from Dr. Letai and Dr. Dalla Favera. These were verified by short tandem repeat (STR) profiling and cultured in standard conditions described in the supplemental methods in supplementary materials.

### 4.2. BH3 Profiling

We used the iBH3 profiling method described previously [44]; originally by Ryan et al. [64]. We thawed 5–10 million cells and stained them with antibodies to the following cell surface markers: CD3, CD19, CD5 (for CLL/SLL only) and CD4, CD8, CD14 (for peripheral blood mononuclear cells only). We exposed cells to digitonin followed by synthetic BH3 peptides that selectively bind specific anti-apoptotic proteins (Figure 1B) and measured cytochrome c release as a read out for mitochondrial apoptosis. We also used the drugs MS1 (MCL1 inhibitor), WEHI-539 (BCLXL inhibitor), ABT-737 (BCL2, BCLXL, and BCLW inhibitor) and venetoclax (BCL2 inhibitor) to further characterize the presence of anti-apoptotic proteins present in cells. Selected drug/peptide concentrations are based upon previously established protocols and applied in various doses where appropriate. We acquired the data on an LSR Fortessa cytometer (BD Biosciences, San Jose, CA, USA) using DIVA (BD Biosciences) software. Our gating strategy is illustrated in Figure S1A. We normalized the data to the DMSO, positive control for cytochrome c retention (i.e., intact mitochondria) and used alamethecin as our positive control for cytochrome c release (MOMP). See supplemental methods in supplementary materials for further description of BH3 profiling and determination of apoptotic block thresholds.

### 4.3. Dynamic BH3 Profiling (DBP)

Cells were incubated for 17 h (h) with 1% PBS, 1% DMSO, or one of the following drug concentrations: 1.2 µM of vincristine, 2 µM of mafosphamide, 50 µM of bendamustine, 10 µM of doxorubicin, 0.5 µM of dexamethasone, 1 µg/mL of vinblastine, 10 ng/mL of monomethyl auristatin E (MMAE). To assess the effect of the drugs on the priming of the cells, and the cells’ sensitivities to venetoclax, we measured the responses to both 1 µM of PUMA peptide and 1 µM of venetoclax in vehicle-treated and drug-treated cells. We then subtracted the untreated response from the treated response to obtain a measure of the change in responses. We performed dynamic BH3 profiling after a 17 h exposure to drugs and measured the levels of anti-apoptotic proteins and MYC by Western blot (see supplemental methods in supplementary materials).

### 4.4. Immunofluorescence

Patient tissue was preserved in formalin-fixed paraffin-embedded blocks that were cut at 4 μM, placed on SuperFrost/Plus slides (VWR, Radnor, PA, USA), and dried overnight at 37 °C. The slides underwent a double immunofluorescence stain for PAX5 and either BIM, BID, BAX, BAK, MCL1, or BCL2. After deparaffinization and hydration, antigen retrieval was carried out in a TRIS/EDTA pH 9.0 buffer for 20 min in a pressure cooker. The slides were blocked with 10% donkey serum for 30 min and then incubated overnight with primary antibody for PAX5 (1:100, Abcam, Cambridge, UK, ab211293) and for the protein of interest at the following dilutions: BIM (1:25, Abcam, ab32158), BID (1:100, Santa Cruz Biotechnology, Dallas, TX, USA, sc-373939), BAX (1:100, Abcam, ab32503), BAK (1:50, Abcam, ab32371), MCL1 (1:50, Abcam, ab32087), or BCL2 (1:50, Abcam, ab32124). After the removal of the primary antibodies, the slides underwent a 1-h secondary antibody incubation with rat AF594 (Invitrogen, Carlsbad, CA, USA, A21209, 1:250) and mouse/rabbit AF647 (Invitrogen, A21235/A21245, 1:500). Finally, the slides were incubated for 15 min with DAPI (Invitrogen, D1306) and mounted with coverslips using prolong gold antifade mountant (Invitrogen, P10144). Imaging was carried out using a Zeiss (Oberkochen, Germany) Axio scan Z1 florescence slide scanner at 20× magnification. Image analysis was carried out using Qupath software for quantitative pathology and bioimage analysis [65]. DAPI was used to identify all cells and then cell intensity for the protein of interest was gated on Pax5+ cells, which were determined as the highest 50% of AF594 expressing cells.

## 5. Conclusions

BCL2 is an attractive target for NHL patients given its role in cell survival and patient prognosis. While clinical trials for venetoclax have shown promising results, there is a wide range of responses to single-agent therapy. Our study helps to highlight potential mechanisms by which malignant cells survive, even after the inhibition of BCL2. Notably, MCL1 has been implicated in previous studies of NHL cell lines as an additional pro-survival protein, important for avoiding apoptosis. This is supported by our analysis of primary patient samples, where the dual inhibition of MCL1 and BCL2 was effective in the majority. While targeting both directly may currently prove to be difficult therapies that affect MCL1 levels outside of direct inhibition may provide an avenue to increase venetoclax effectiveness in NHL. Our cohort also highlights a previously underreported group in NHL that has severe pro-apoptotic defects. While these samples were few, they all displayed resistance to peptides and inhibitors targeting anti-apoptotic proteins. Patients displaying this BH3 profile are unlikely to respond to therapies involved in activating mitochondrial apoptosis. The mechanism of this defect remains to be seen, as it is unlikely to be a result of reduced protein expression (potentially the case in class A) or genetic aberrations to the genes responsible for their production. In summary, the BH3 profiling of patient samples is a fast and effective technique that could identify patients who may benefit from a specific targeted therapy. The continued expansion of our cohort via BH3 profiling and the discovery of the source of class B, pro-apoptotic defects can help inform future clinical trial and patient management decisions for certain subclasses of NHL patients.

## Data Availability

The data presented in this study are available on request from the corresponding author. The data are not publicly available due to privacy restrictions.

## Supplementary Materials

The following are available online at https://www.mdpi.com/2072-6694/13/5/1002/s1, Figure S1: BH3 profiling, Figure S2: Normal B Cell Response, Figure S3: Full BH3 profiling results for all non-malignant cell types tested, Figure S4: Full BH3 profiling results for all NHL samples tested, Figure S5: Expression of BAK, BID, and BCL2 in DLBCL BH3 profiles, Figure S6: Immunofluorescence of BAK, BID, and BCL2, Figure S7: Combination of WEHI-539 and Venetoclax in FL and DLBCL, Figure S8: Expression of BIM in vincristine treated NHL cell lines, Table S1: Characteristics of all 124 lymphoma samples used for BH3 profiling, Table S2: Mean intensity values for immunofluorescence staining of PAX5+ cells.
