# Supplementary material for: Apoptotic Blocks in Primary Non-Hodgkin B Cell Lymphomas Identified by BH3 Profiling
Source: Cancers (Basel). 2021 Feb 28;13(5):1002. doi: 10.3390/cancers13051002 (PMC7957722; doi:10.3390/cancers13051002)
Supplement: Supplementary file 1 [file cancers-13-01002-s001.pdf]

Supplementary Materials: Apoptotic Blocks in Primary Non-Hodgkin B Cell Lymphomas Identified by BH3 Profiling

Ryan N. Rys, Claudia M. Wever, Dominique Geoffrion, Christophe Goncalves, Artin Ghassemian, Eugene Brailovski, Jeremy Ryan, Liliana Stoica, Josée Hébert, Tina Petrogiannis-Haliotis, Svetlana Dmitrienko, Saul Frenkiel, Annette Staiger, German Ott, Christian Steidl, David W Scott, Pierre Sesques, Sonia del Rincon, Koren K. Mann, Anthony Letai and Nathalie A. Johnson

Supplemental Methods

Patient Sample Preparation

Patient samples that were collected in liquid form (blood or other fluid) were diluted in phosphate buffered saline (PBS) (total of 3–6 mL) and were gently layered onto 3 mL of ficoll medium, spun with no brake at 800 G for 20 mins, and the opaque interface layer containing the peripheral blood mononuclear cells (PBMCs) was isolated. Tissue-derived patient samples were disaggregated using the GentleMACS C tube system according the manufacture's procedures (Miltenyi Biotec, Bergisch Gladbach, Germany). Isolated cells of interest were transferred to freezing medium (10% dimethyl sulfoxide (DMSO) and 90% fetal bovine serum (FBS)) and frozen in cryovials at −80°C overnight before being transferred to liquid nitrogen for long-term storage.

BH3 Profiling- Additional Information

We used the LIVE/DEAD™ Fixable Aqua Dead Cell Stain Kit (Thermo Fisher Scientific, Waltham, MA, USA, L34957) to assess viability, and used only live cells in our analysis. After viability staining, we stained with the following antibodies for flow cytometry, all obtained from BD biosciences (San Jose, CA, USA): CD3 BV786 (clone SK7, catalog number 563800), CD19 (clone H1B19, catalog number 561295), CD5 (clone UCHT2, catalog number 555353, used only for CLL/SLL), CD14 (clone MΦP9, catalog number 563744, used only for PBMCs), CD4 (clone RPA-T4, catalog number 560345, used only for PBMCs), CD8 (clone SK1, catalog number 641400, used only for PBMCs). Antibodies were incubated with the samples for 30mins at 4 °C, in PBS + 2% FBS. Fc block (BD biosciences, catalog number 564220) was used prior to staining PBMCs.

We defined a class B block as having a response of <30% to 100 μM BIM peptide, a threshold that was selected because it was more than 2.5 standard deviations below the normal distribution of cytochrome c release in normal B cells in response to BIM or PUMA (Figure S2 and Figure 2). A class A block was defined as having response of ≥ 30% to 100 μM BIM but < 30% to 100 μM PUMA peptide, a promiscuous sensitizer peptide that binds to all the anti-apoptotic proteins. Class C blocks were identified as having responses of >30% to both BIM and PUMA.

Cell Culture

Suspension cells were incubated in culture flasks at 37 °C. SU-DHL-4, SU-DHL-6, SU-DHL-8, and SU-DHL-10 cell lines were cultured in 10% FBS in RPMI-1640; OCI-Ly1 and OCI-Ly8 cells were cultured in 0.1% β-mercaptoethanol added to 10% FBS in RPMI-1640 medium; TOLEDO were cultured in 20% FBS in RPMI 1640 medium. 2.5 mL of 10,000 IU penicillin/10,000 μg/mL streptomycin solution was added to every 500 mL stock bottle of cell medium to inhibit bacterial growth. All cell lines tested negative in the past 6 months for mycoplasma using a PCR detection from ABM (Richmond, BC, Canada, Cat. G238). Cell lines were verified using short tandem repeat analysis (TCAG, SickKids, Toronto, ON, Canada).

MTT Assay Protocol

The TACS® MTT Cell Proliferation Assay kit was purchased from Trevigen® (Gaithersburg, MD, USA). Cells were plated into 96-well plates at 100,000 cells/ well for 17 hours. Cells were counted using a hemocytometer, harvested, centrifuged at 300 G for 5 minutes, and resuspended in fresh medium immediately prior to plating at a cell concentration of 1 million cells/mL. 20× drug solutions were prepared by diluting a 1000× drug stock solution in DMSO by 50-fold in the appropriate cell medium. After incubation, 10 μL of MTT solution was added to each well and plates were incubated again for between 2–4 hours or until visible appearance of violet formazan precipitate. The precipitate was then solubilized by addition of 100 μL of detergent solution and left at 37 °C overnight. Absorbance was measured at 570 nm with a reference wavelength at 700 nm. Absorbance measurements were blank-corrected and % viability was normalized to the average of untreated cells for constructing viability curves and to DMSO for correlating to BH3 profiles.

Percentages of viable cells after 17-hour treatment with venetoclax were calculated by normalizing to DMSO vehicle representing 100% viability. Spearman's correlation analysis was performed using GraphPad Prism to determine the correlations between MTT vs. % cytochrome C release. Statistical significance was calculated as $p < 0.05$ according to Student's t-test (two-tailed).

Western blotting

Western blots were performed according to standard procedures as described previously (42), with the added usage of the primary antibody against BIM (catalog number ab32158, abcam, Cambridge, UK), Vinculin (catalog number 4650S, Cell Signaling Technology, Danvers, MA, USA), or actin (clone I-19, catalog number SC1616, Santa Cruz Biotechnology, Dallas, TX, USA). Western blots were imaged using an Azure (Dublin, CA, USA) c600 gel imaging system. Protein levels were calculated the following way: Protein level = Optical density for the Protein/Optical density for the loading control.

Statistics

We determined if there were any significant differences between lymphoma subtypes and normal cells by comparing the percentage of cells releasing cytochrome c upon exposure to each peptide using a one-way ANOVA with Tukey multiple comparison. Two-way ANOVA was used to compare peptide exposure/subtype to cytochrome c release in normal B cells. The differences between the levels of protein expression by immunofluorescence, western blot and the changes in priming upon exposure to chemotherapy were determined using Welch's t test. Spearman's correlation analysis was performed to determine correlations where appropriate. A p value of < 0.05 was considered significant. Statistical analysis was performed in Prism7/8 (GraphPad Software, Inc., San Diego, CA, USA) and SPSS (version 23, IBM, Armonk, NY, USA).

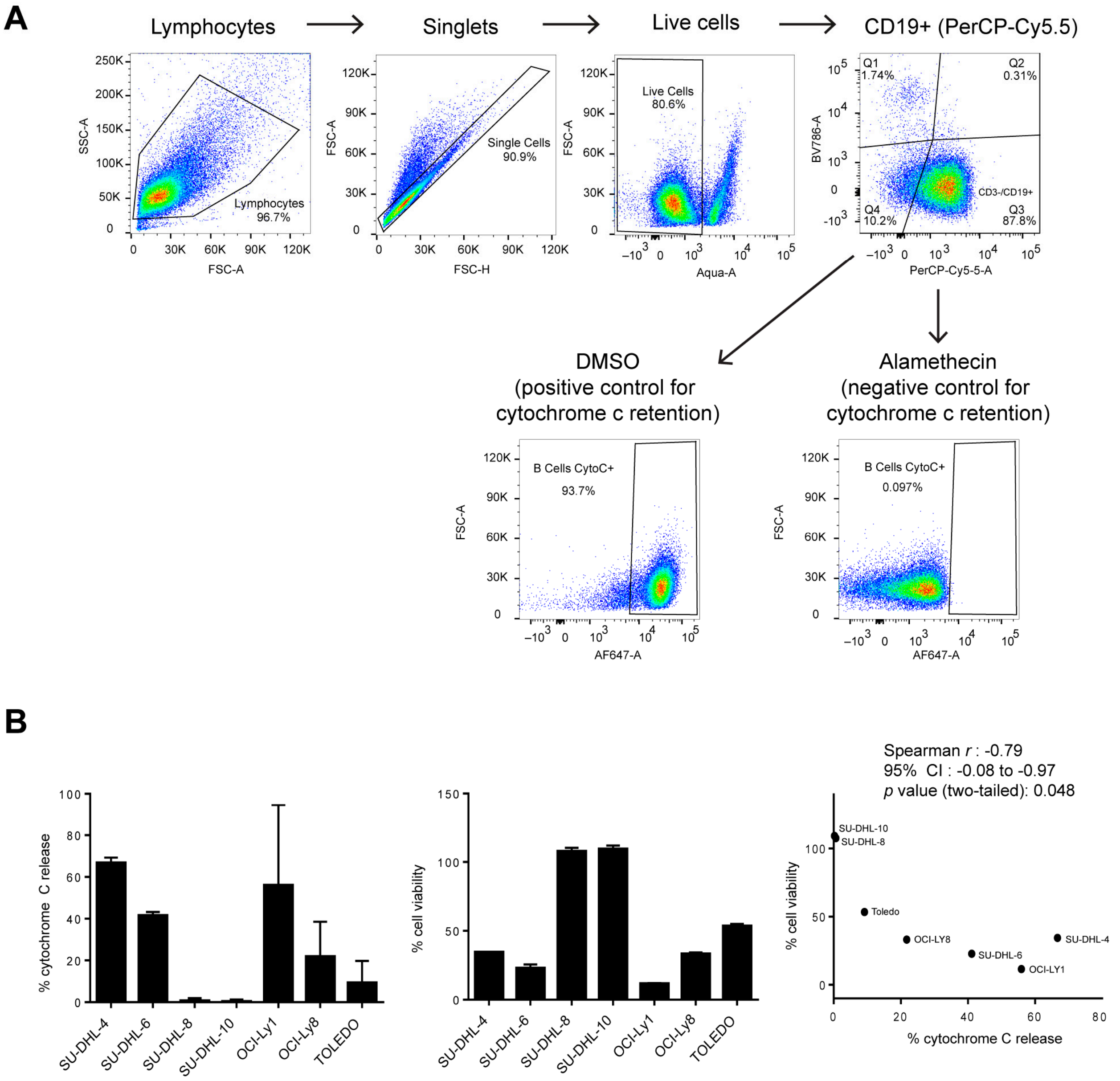

Figure S1. BH3 profiling. (**A**) Gating strategy used to identify population of interest for BH3 profiling analysis. Cytochrome c stains positively when retained in the cell. Therefore, Alamethecin is a negative control for cytochrome c retention and a positive control for cytochrome c release. DMSO acts as the inverse control: positive for retention and negative for release. (**B**) Left panel: cytochrome C release of seven DLBCL cell lines in response to venetoclax as measured by BH3 profiling. Center panel: Cell viability after 17-hour treatment with 2.4 µM venetoclax. Viability was normalized to DMSO vehicle as 100% viability and cell medium blanks as 0%. Right panel: Correlation of cell viability after treatment with venetoclax for 17 hours and cytochrome C release by BH3 profiling. There is a significant negative correlation between the two variables, indicating that increasing cytochrome C release in response to venetoclax during BH3 profiling correlates significantly to decreasing cell viability in response to venetoclax treatment.

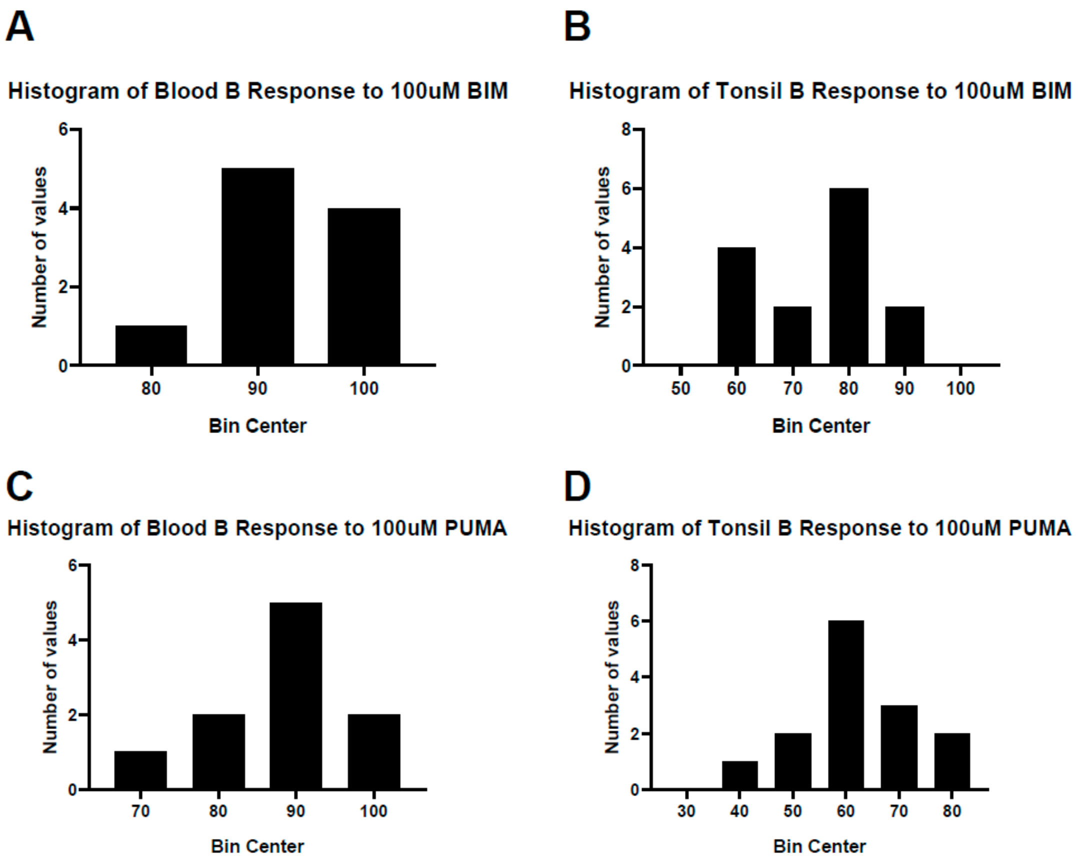

Figure S2. Normal B Cell Response. Histograms showing the range of responses for normal B cells to 100 µM BIM (**A,B**) and 100 µM PUMA (**C,D**) in PB and LN. These responses were used to determine the threshold for class A/B samples outlined in the methods. PB shows less variation and higher overall responses.

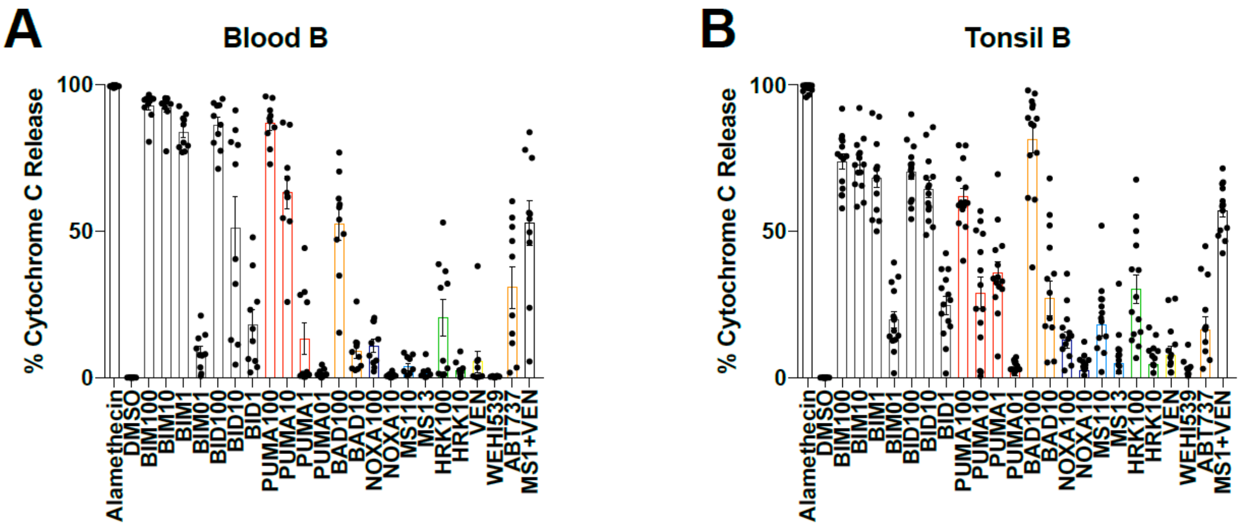

Figure S3. Full BH3 profiling results for all non-malignant cell types tested. (**A**) BH3 profiling of peripheral blood (PB) B cell controls (n = 10) showing % of the cells undergoing cytochrome c release on the Y axis after exposure to each drug/peptide on the X axis. Cells exhibit a Class C response with multiple anti-apoptotic protein dependencies. (**B**) BH3 profiling of tonsil B cell (n = 14). Tonsillar samples are similar to PB controls but are slightly less primed and more dependent on MCL1.

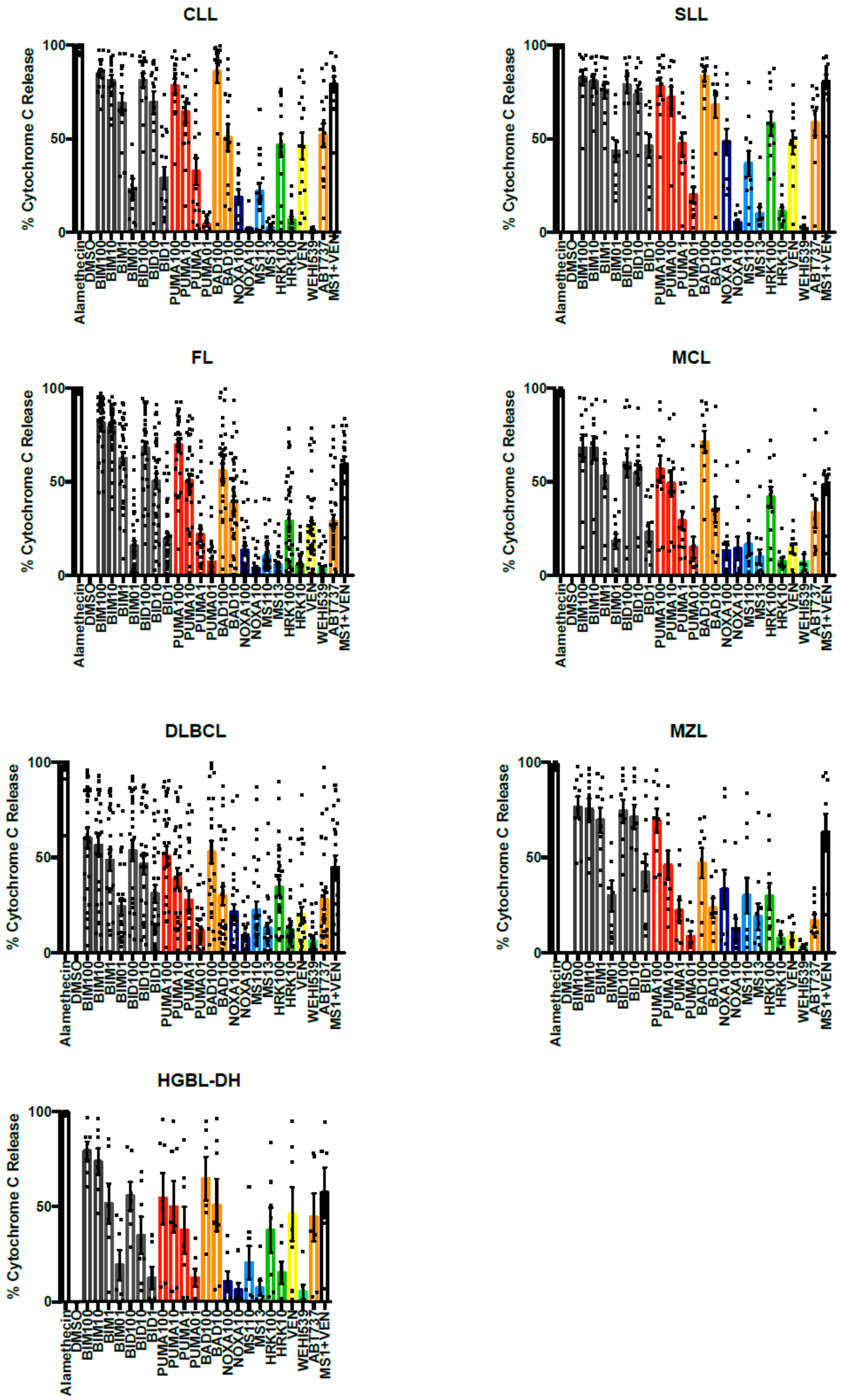

Figure S4. Full BH3 profiling results for all NHL samples tested. Full BH3 profiles for all NHL subtypes tested. See supplemental table 1 for full summary of samples used for analysis.

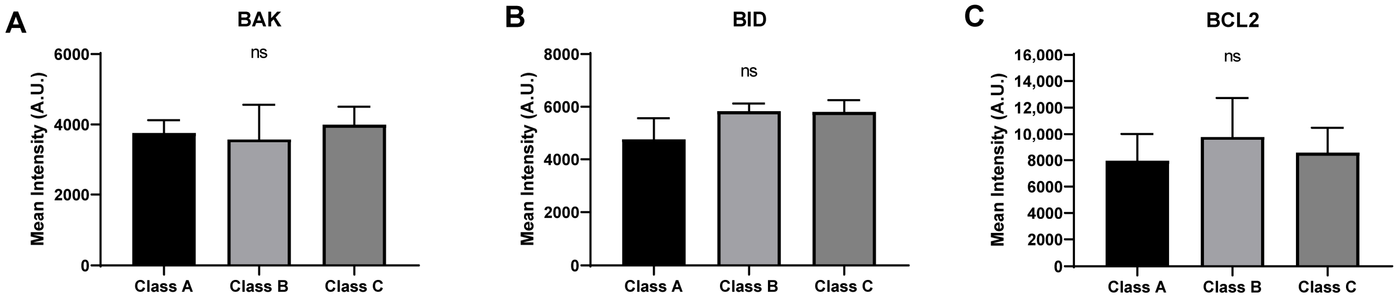

Figure S5. Expression of BAK, BID, and BCL2 in DLBCL BH3 profiles. Quantification of mean pixel intensity across all classes of apoptotic block in BAK (**A**), BID (**B**), and BCL1 (**C**). Samples were analyzed using Qupath software (see methods) and *t*-test with Welch's correction was used to compare columns, bars represent the mean ± standard error of the mean (SEM). No significant differences in expression intensity were seen in these proteins across all classes. ns, non-significant.

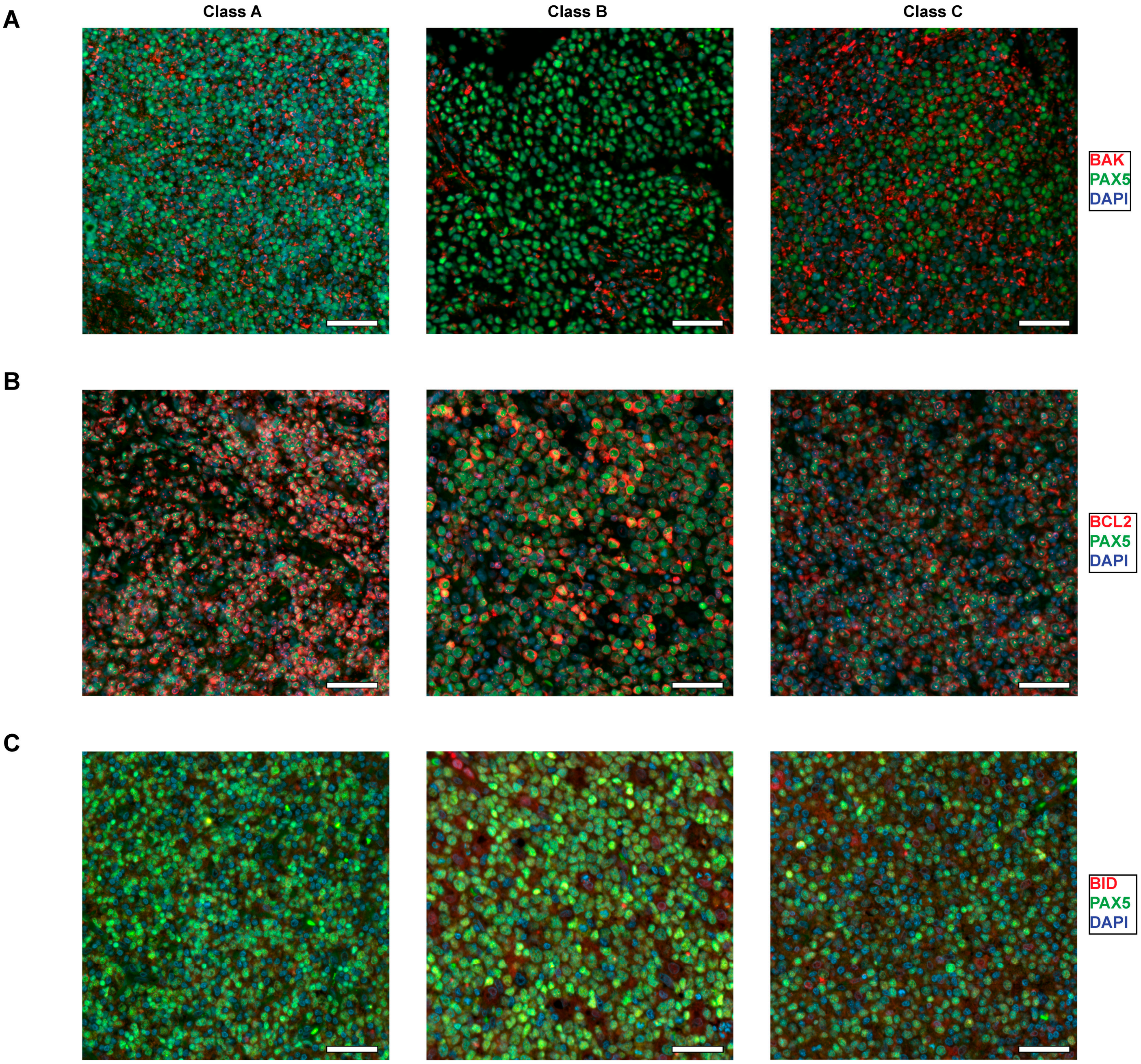

Figure S6. Immunofluorescence of BAK, BID, and BCL2. Representative merged IF stains at 20× for DAPI (blue), PAX5 (green, AF594) and BAK/BID/BCL2 (red, AF647) in class (**A**), (**B**), and (**C**) samples. Scale bars represent 50 μm.

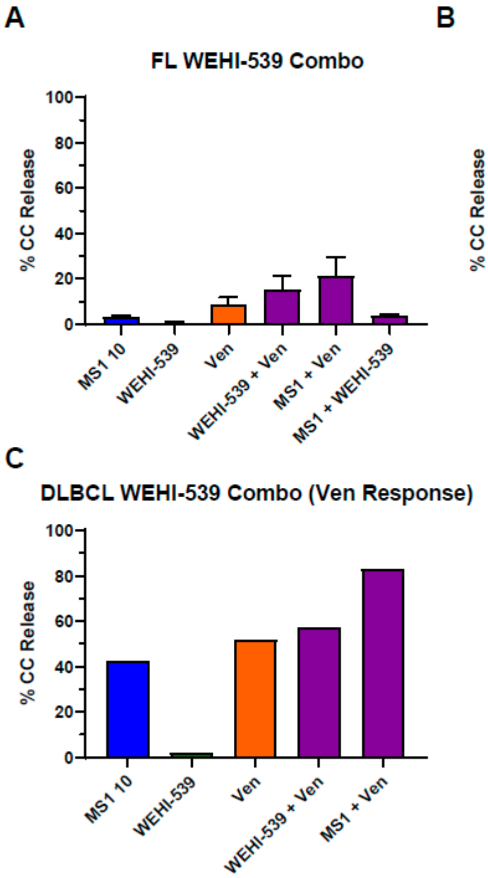

Figure S7. Combination of WEHI-539 and Venetoclax in FL and DLBCL. A subset of FL (*n* = 7) and DLBCL (*n* = 4) samples also included combination of either 10 µM MS1 or 1 µM WEHI-539 and 1 µM Venetoclax during BH3 profiling. Error bars represent standard error of mean (SEM) (**A**) Summary BH3 profiles of FL samples also incubated with combination targeting of BCLXL and BCL2. Only marginal increases in cytochrome c release are seen with the additional targeting of BCLXL by WEHI-539. Additional samples in this group (*n* = 3) were also treated with combination MCL1 and BCLXL targeted inhibitors (MS1 and WEHI-539, respectively). These samples showed no response and implicated BCL2 as the primary protein responsible for cell survival. (**B**) Summary BH3 profiles of DLBCL samples also incubated with combination targeting of BCLXL and BCL2. Only marginal increases in cytochrome c release are seen with the additional targeting of BCLXL by WEHI-539. (**C**) Specific DLBCL sample with a strong response to venetoclax. Here the targeting of BCLXL provides little benefit to cytochrome c release and is substantially less than combination treatment with MS1.

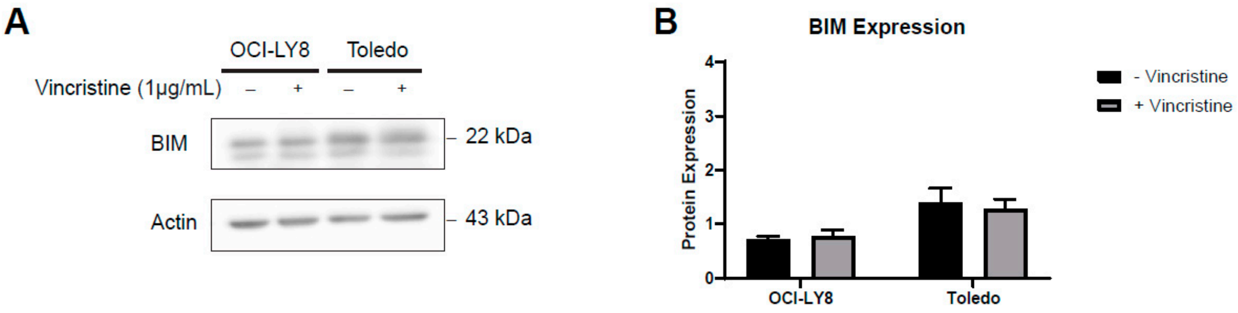

Figure S8. Expression of BIM in vincristine treated NHL cell lines. (**A**) Representative western blot of BIM expression after 17 h treatment with 1 µg/mL vincristine. (**B**) Quantification of BIM expression via western blot (*n* = 3). No difference in expression was seen between treated and untreated samples, as analyzed by multiple paired *t*-tests. Error bars represent standard error of mean (SEM).

Unadjusted Western Blots (Figure 7 Panel C OCI-LY8)

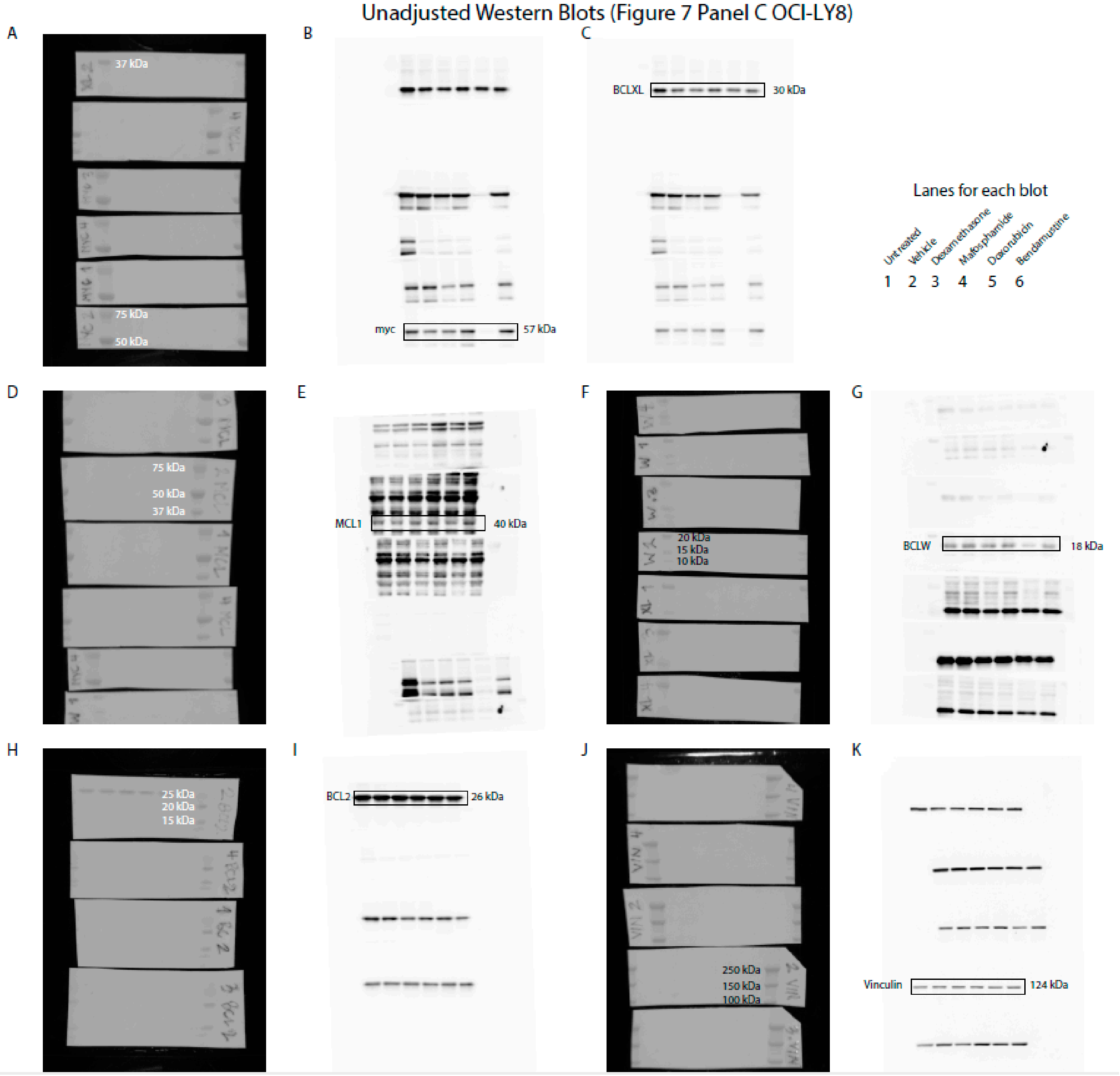

Unadjusted Western Blots (Figure 7 Panel C Toledo)

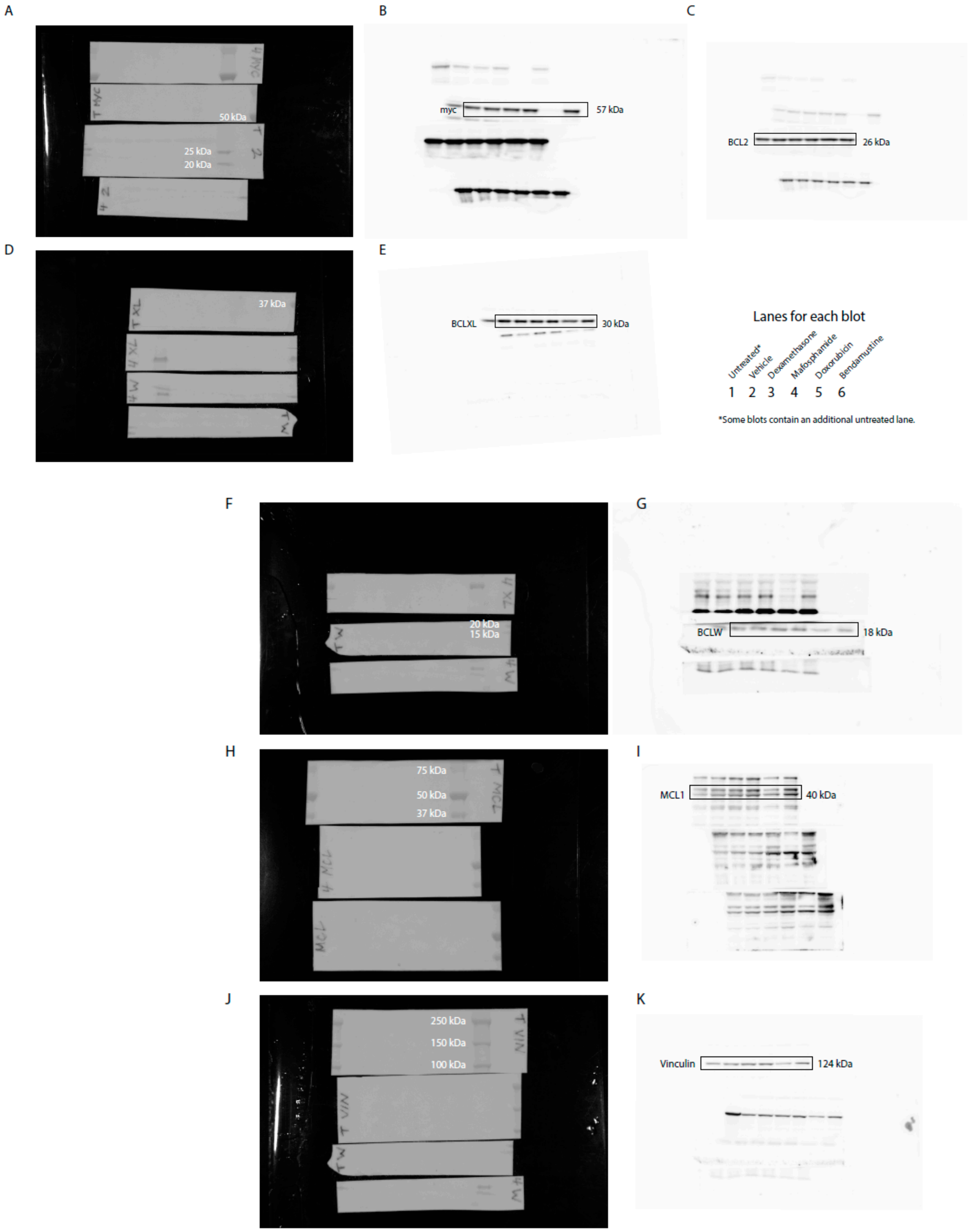

Unadjusted Western Blots (Figure 7 Panel D)

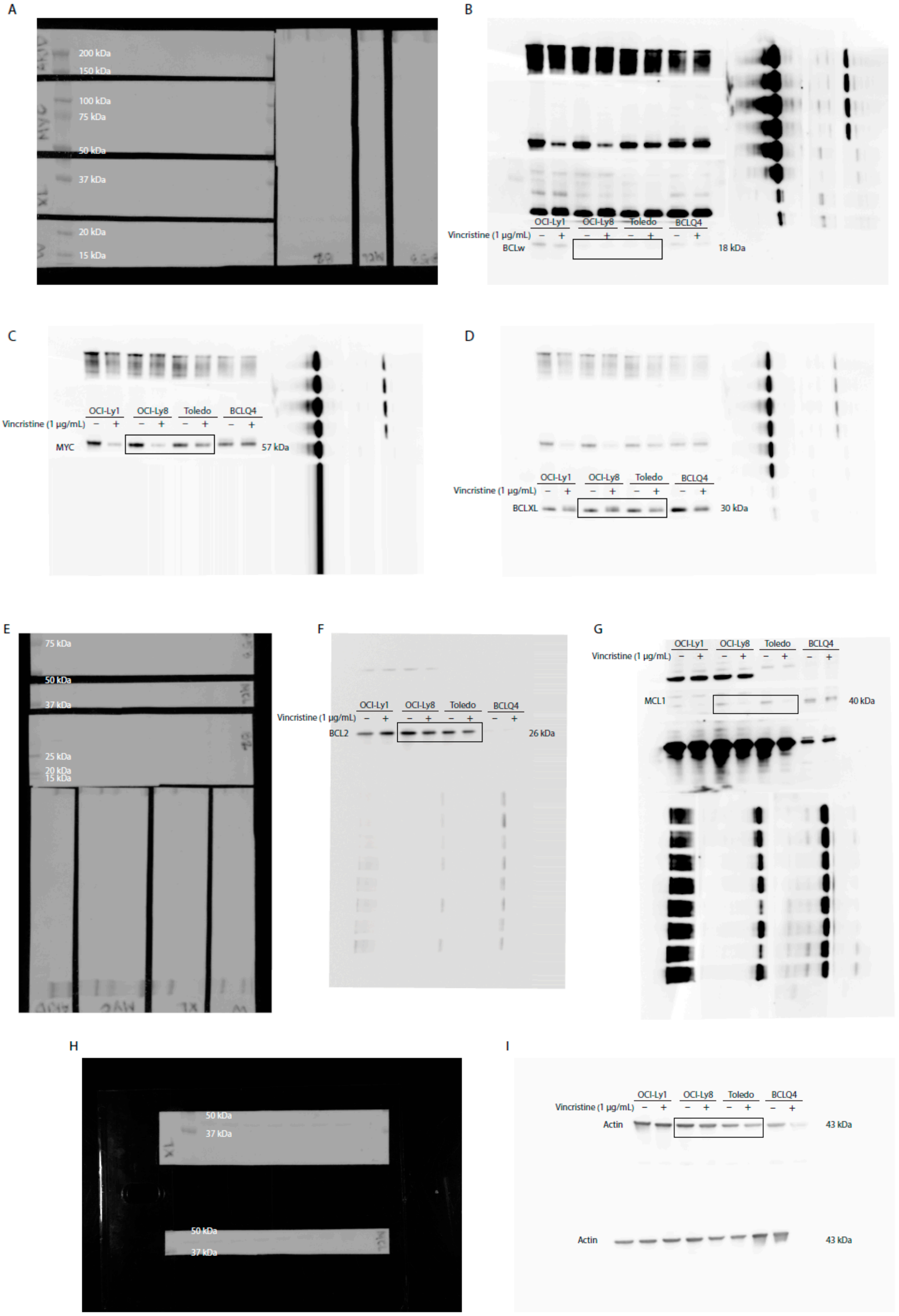

Detail information about Figure 7.

Figure S8: Unadjusted Western Blot

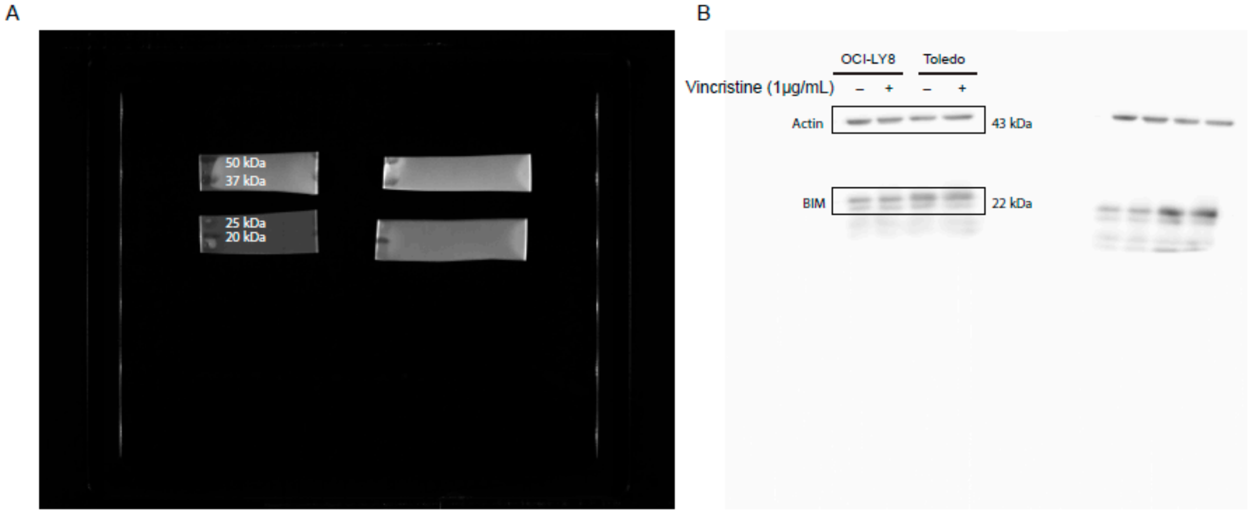

Detail information about Figure S8. The provided full western blot images contain marker images combined with the respective exposed blot. These blots were done at the same time as many other blots concerning other cell lines and experiments not included in this manuscript. As such, only the markers corresponding to the blots relevant to this publication have been annotated. Many blots were cut and imaged together, resulting in the marker and exposed images seen here. Weight markers are annotated based on the use of a dual color protein ladder (Bio-Rad, #1610374). Full images were submitted, even though they contain additional information not pertinent to this study, for reasons of transparency and providing full un-edited blots. Black boxes in each image highlight the lanes used in the respective figure indicated in the file (either Figure 7C,D, or S8).

Table S1. Characteristics of all 124 lymphoma samples used for BH3 profiling.

Characteristics	Number of Cases
CLL/SLL	27 (16 blood, 11 LN)
Follicular lymphoma (FL)	38 (LN)
Marginal zone lymphoma (MZL)	10 (LN)
Mantle cell lymphoma (MCL)	12 (LN)
HGBL-DH	7 (1 LN, 6 fluid)
DLBCL*	30 (26 LN, 4 fluid)
BCL2 protein > 50% cells	
Positive	19
Negative	8
Not available	3
BCL2 translocation	
Present	3
Absent	19
Not available	8
Cell of origin	
GCB	11
Non-GCB	16
Not available	3

Lymphoma cells were obtained from lymph nodes (LN) that were disaggregated into a cell suspension or cells were already in single cell suspension derived from fluid samples that included peripheral blood, cerebrospinal fluid, pleural fluid, ascites and bone marrow. Cell of origin was determined by immunohistochemistry using Hans criteria except for 4 DLBCL samples from the British Columbia Cancer Agency where nanostring was used. The one unclassifiable sample was assigned to the non-GCB group. *One patient with high-grade B cell lymphoma without concurrent *MYC* and *BCL2* translocations was included in the DLBCL cohort, sample taken at the time of diagnosis (LN). Abbreviations: LN, lymph node; HGBL-DH, high grade B cell lymphoma double hit, with translocations in MYC and BCL2; DLBCL, diffuse large B cell lymphoma; CLL, chronic lymphocytic leukemia; SLL, small lymphocytic lymphoma; GCB, germinal center B cell lymphoma.

Table S2. Mean intensity values for immunofluorescence staining of PAX5+ cells.

Class	BAX Mean Intensity (A.U.)	BIM Mean Intensity (A.U.)	MCL1 Mean Intensity (A.U.)	BCL2 Mean Intensity (A.U.)	BAK Mean Intensity (A.U.)	BID Mean Intensity (A.U.)
A	3853.288417	1374.648764	1091.10804	5903.325116	3398.310483	5573.286071
A	4472.266827	1258.928672	1015.728332	10023.18012	4124.485058	3978.781223
B	7098.61491	9941.187506	2240.660679	14709.78703	2019.337381	6476.903207
B	5663.862758	10344.48309	1770.131158	14601.15274	2436.199947	5347.320627
B	10129.45186	5841.492441	3258.356392	7003.465439	5521.539065	6601.324827
B	6285.105063	5258.880078	1989.559763	N/A	6339.458534	5469.620352
B	4417.091844	4276.640875	1360.384019	2839.007864	1579.364029	5278.815957
C	10121.14511	19828.45307	2326.923568	17134.13658	3957.462731	4675.663653
C	1796.305482	3839.670857	1599.298839	4141.41242	1875.73769	6049.616008
C	5556.709505	18974.15932	2543.152245	5029.490361	3289.689541	5781.86078
C	7413.787418	5760.20464	3538.071734	8811.855963	5670.484968	4504.526667
C	10668.02037	10522.29172	2690.25151	9510.976622	5689.042839	5305.112272
C	6117.23331	11526.97544	3597.869027	N/A	3654.310946	7910.982896
C	8247.859508	12880.31174	3229.2039	N/A	N/A	N/A
C	8084.438601	4547.557853	2916.041703	6871.081087	3846.662773	6457.956317

Mean intensity was calculated by Qupath image analysis software. Cell intensity for protein of interest was gated on Pax5+ cells, which were determined as the highest 50% of AF594 expressing cells. Values shown are the mean intensity of all Pax5+ cells in a sample analyzed and samples labeled "N/A" indicate either a staining failure and/or lack of sufficient tissue to characterize the expression of stained protein.

© 2021 by the authors. Licensee MDPI, Basel, Switzerland. This article is an open access article distributed under the terms and conditions of the Creative Commons Attribution (CC BY) license (http://creativecommons.org/licenses/by/4.0/).
